# MicroRNA profiling of rhesus macaque embryonic stem cells

**DOI:** 10.1186/1471-2164-12-276

**Published:** 2011-05-31

**Authors:** Zhenghua Sun, Qiang Wei, Yanfeng Zhang, Xiechao He, Weizhi Ji, Bing Su

**Affiliations:** 1 State Key Laboratory of Genetic Resources and Evolution, Kunming Institute of Zoology, Chinese Academy of Sciences, Kunming 650223, China; 2 Department of Reproduction and Development, Kunming Institute of Zoology, Chinese Academy of Sciences, Kunming 650223, China; 3 Kunming Biomed International and National Engineering Research Center of Biomedicine and Animal Science, Kunming 650051, China; 4 Graduate School of Chinese Academy of Sciences, Beijing 100049, China

## Abstract

**Background:**

MicroRNAs (miRNAs) play important roles in embryonic stem cell (ESC) self-renewal and pluripotency. Numerous studies have revealed human and mouse ESC miRNA profiles. As a model for human-related study, the rhesus macaque is ideal for delineating the regulatory mechanisms of miRNAs in ESCs. However, studies on rhesus macaque (r)ESCs are lacking due to limited rESC availability and a need for systematic analyses of fundamental rESC characteristics.

**Results:**

We established three rESC lines and profiled microRNA using Solexa sequencing resulting in 304 known and 66 novel miRNAs. MiRNA profiles were highly conserved between rESC lines and predicted target genes were significantly enriched in differentiation pathways. Further analysis of the miRNA-target network indicated that gene expression regulated by miRNAs was negatively correlated to their evolutionary rate in rESCs. Moreover, a cross-species comparison revealed an overall conservation of miRNA expression patterns between human, mouse and rhesus macaque ESCs. However, we identified three miRNA clusters (miR-467, the miRNA cluster in the imprinted Dlk1-Dio3 region and C19MC) that showed clear interspecies differences.

**Conclusions:**

rESCs share a unique miRNA set that may play critical roles in self-renewal and pluripotency. MiRNA expression patterns are generally conserved between species. However, species and/or lineage specific miRNA regulation changed during evolution.

## Background

ESCs are derived from the inner cell mass (ICM) of blastocyst-stage embryos [1,2]. Self-renewal and pluripotency enable ESCs to be a renewable and versatile model to study developmental biology. Moreover, ECSs have potential applications in regenerative medicine. Rhesus macaques (*Macaca mulatta*) are a well-studied primates, and with genetic and physiological similarities to humans, rhesus macaques have become an ideal model for ESC-based therapies [3]. However, the study and application of rESCs are lacking compared with those of mouse and human ESCs due to limited rESC line availability and a need for systematic analyses of fundamental rESC characteristics.

MiRNAs are small endogenous non-coding transcripts (~19-25 nt) with diverse roles in development, differentiation and oncogenesis. MiRNAs bind to complementary sites within cognate mRNA 3' UTRs, resulting in degradation, deadenylation or translational repression, which provide a crucial level of post-transcriptional regulation [4]. Moreover, tissue- and cell type-specific miRNA expression patterns have been described [5-9], which elucidate various miRNA functions in specific conditions. MiRNAs also play important roles in ESCs as demonstrated by deletion of Dicer or DGCR8 in mouse ESCs resulting in proliferation and differentiation defects [10-12]. Previous studies of miRNA expression patterns in mouse and human ESCs have revealed a unique miRNA set that is distinct from other cell types and tissues [6,13,14]. Several miRNAs preferentially expressed in human and mouse ESCs, and down-regulated in differentiated cells are key regulators of 'stemness' [15-19]. However, the miRNA expression profile of rESCs is unknown.

ESC lines derived from the same species may contain distinct miRNA profiles and share only a small number of miRNAs [20]. This observation is likely caused by various ESC culture conditions rather than inherent genetic variation within embryos used for ESC derivation [21,22]. Another factor is the use of numerous analyses for detecting miRNA patterns due to the limited resolution of techniques such as microarray analysis [23]. However, recent advancements in next-generation sequencing technology provide an ideal tool for analyzing the miRNA transcriptome with high resolution to identify novel miRNAs [24].

In this study, we isolated and characterized three rESC lines, and performed miRNA profiling using Solexa sequencing. Our miRNA study of rESCs and cross-species comparison may assist future studies for understanding and modulating ESC regulatory networks.

## Results

### Isolation and characterization of rESC lines

**Figure 1:**
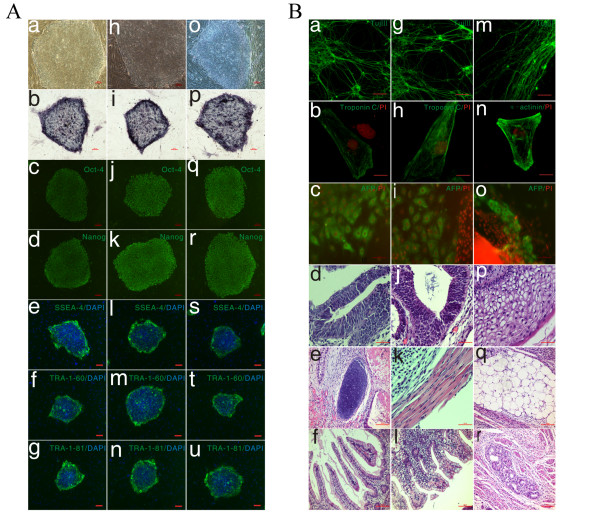
**'Stemness' and pluripotency of rESC lines**. (A): (a-g): IVF1.2; (h-n): IVF3.2; (o-u): IVF3.3; (a, h, o): Phase-contrast micrograph of an ESC colony on mEFs; (b, i, p): Alkaline phosphatase staining; (c, j, q): Oct-4; (d, k, r): Nanog; (e, l, s): SSEA-4; (f, m, t): TRA-1-60; (g, n, u): TRA-1-81; (B): (a-f): IVF1.2; (g-l): IVF3.2; (m-r): IVF3.3; (a, g, m): Neuronal marker Tuj III expression (green); (b, h) Cardiomyocyte marker α-cardiac actinin (green); (n): Cardiomyocyte marker Troponin C (green); (c, i, o) Endoderm marker α-fetoprotein (AFP, green); (d, j): Neural tube; (p): Squamous epithelium; (e): Cartilage; (k): Muscles; (q): Adipose; (f, l): Intestinal epithelia; (r): Gland. Scale bars: (A: a-u, 100 μm; B: a, g, m, c, i, o, 100 μm; b, h, n, 20 μm; d-f, j-l, p-r, 50 μm).

Thirteen expanded rhesus macaque blastocysts with a prominent ICM were selected by immunosurgery and 11 ICMs were isolated and plated onto feeder cells. ICMs attached to feeder cells within 48 h and three ESC-like ICM outgrowths appeared after 7-8 days. ICM outgrowths were manually dissociated into 4-6 smaller clumps using a microscalpel, excised from feeder cells and replated onto fresh mouse embryonic fibroblasts (mEFs). Clones with distinct boundaries and high nuclear to cytoplasm ratios were selected for further propagation. Three rESC lines were established and designated as IVF1.2, IVF3.2 and IVF3.3. IVF3.2 and IVF3.3 were derived using the same sperm and oocyte donors. rESCs shared common morphologies with other primate ESCs such as being flat with a distinct boundary against feeder cells. Cells showed high nuclear to cytoplasm ratios and prominent nucleoli (Figure 1A). IVF1.2 and IVF3.3 were cultured for >60 passages and IVF3.2 for >80. Pluripotency markers were highly expressed in all rESC lines including Oct-4, Nanog, SSEA-4, TRA-1-60 and TRA-1-81 (Figure 1A). Other crucial transcription factors, such as Sox-2 and Rex-1, were also detected by RT-PCR (data not shown).

All rESC lines showed strong alkaline phosphatase activity (Figure 1A) and could spontaneously differentiate into cell lineages from the three embryonic germ layers. After 2 days in suspension culture, rESC clones formed embryoid bodies (EBs). After 5 days, cavities were observed in EBs, which are termed cystic EBs. EBs attached to gelatin-coated dishes and were cultured for 5-7 days until various differentiated cells appeared including neuron-like cells, contractive cardiomyocytes and endoderm-like cells (Figure 1B). Teratomas were formed 6-7 weeks after rESC injection into the hind leg muscles of SCID mice. Histological characterization revealed that ectoderm (neural tube and squamous epithelium), mesoderm (cartilage, muscle and adipose) and endoderm (intestinal epithelia and glands) like structures were present in teratomas (Figure 1B). These results demonstrated that rESC lines could differentiate into all three embryonic germ layers *in vitro *and *in vivo*.

Detailed G-banding analysis revealed that all rESC lines were karyotypically normal with a diploid set of 42 chromosomes (Fig. S1 in Additional file 1), even after long-term culture *in vitro *and repeated freeze-thaw procedures. IVF1.2 and IVF3.3 were derived from male blastocysts (40+XY) and IVF3.2 was derived from a female blastocyst (40+XX).

### Small RNA Sequencing

**Figure 2:**
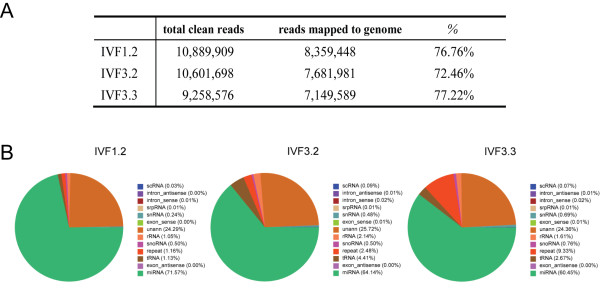
**Summary of small RNA sequencing**. (A): Counts and proportion of mapped tags in total clean reads; (B): Annotation of 13 RNA categories in pie chart for IVF1.2, IVF3.2 and IVF3.3.

Newly established rESCs were tested for purity using Oct-4 staining, which resulted in >95% Oct-4-positive cells (Fig. S2 in Additional file 1). A small RNA library from each cell line was then constructed for sequencing. Small RNA library sequencing yielded 12.66 × 10^6^, 13.12 × 10^6^and 11.57 × 10^6 ^raw reads from IVF1.2, IVF3.2 and IVF3.3, respectively. After filtering, 10.89 × 10^6 ^(IVF1.2), 10.60 × 10^6 ^(IVF3.2) and 9.26 × 10^6 ^(IVF3.3) clean reads (18-30 nt) were obtained. The length distribution of clean reads centered at 22-23 nt, coinciding with the miRNA length range. Sequence alignment with the rhesus macaque genome (rheMac2) using the Short oligonucleotide alignment program (SOAP) [25] demonstrated that 8.36 × 10^6 ^(IVF1.2), 7.68 × 10^6 ^(IVF3.2) and 7.15 × 10^6 ^(IVF3.3) sequences were mapped to the genome (Figure 2A). MiRNAs were the major component of small RNA libraries from rESCs (Figure 2B) being 60% of sequences, which were matched to reference miRNAs in each sample. The proportions of other annotated RNAs was <15%. Notably, ~25% of sequences could not be annotated because of low coverage (6 × ) and relatively poor annotations of the available rhesus macaque genome. Unknown sequences were further analyzed to identify novel miRNAs.

### MiRNA expression profiles of rESC lines

After matching to 466 reference rhesus macaque miRNAs, 326, 329 and 326 known miRNAs were detected in IVF1.2, IVF3.2 and IVF3.3, respectively, with absolute counts ranging from 1-1,702,656 (Table S1 in Additional file 1). Among them, 304 miRNAs were shared by the three pools and remaining miRNAs were expressed at low levels with <10 counts. From a genome-wide perspective, very low miRNAs were likely sequencing artifacts or miRNA machinery byproducts [26]. Therefore, an absolute count of 30 was set for each pool as the minimum threshold for further analyses.

**Figure 3:**
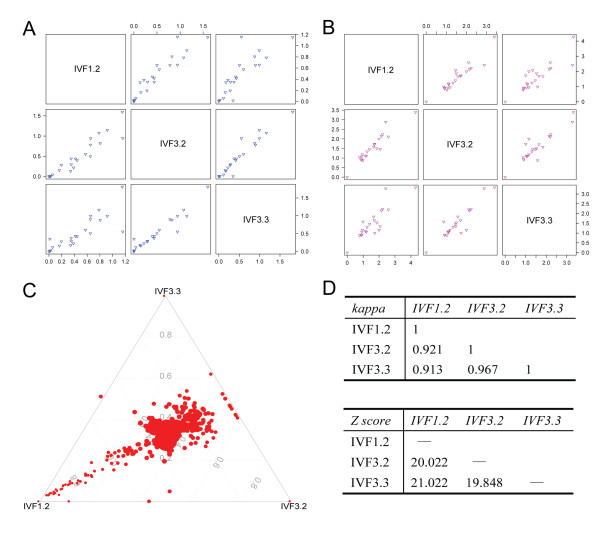
**Consistency of miRNA expression among three rESC lines**. Pairwise comparisons of miRNA clusters between IVF1.2, IVF3.2 and IVF3.3 were performed. Each triangle dot represents an individual pairwise comparison of a miRNA cluster based on variability (A) and CV statistics (B); (C): Trio-comparison of distinct miRNA expression between IVF1.2, IVF3.2 and IVF3.3 based on the differential index. Each point indicates the normalized count of each miRNA; (D): Association analysis of total miRNA expression between IVF1.2, IVF3.2 and IVF3.3 using Kappa statistical analysis and Z-score.

To compare miRNA expression patterns between rESC lines, we evaluated the expression variability (Var) and coefficient of variation (C.V.) of miRNA clusters (Figure 3A, 3B). The results revealed similar expression patterns of miRNA clusters in rESC lines. We also compared the expression of total miRNAs using a differential index (D.I.) and Kappa Statistical analyses. For the D.I., most miRNAs were consistently expressed (Figure 3C), whereas a slight deviation existed in IVF1.2 due to low-level expressed miRNAs. For Kappa Statistical analysis, there was high repeatability between any two cell lines (Figure 3D). Taken together, miRNA expression patterns in rESC lines were highly consistent.

### Identification of novel miRNAs

We predicted novel miRNA genes in unannotated sequences using Mireap (http://sourceforge.net/projects/mireap/). Briefly, according to the characteristic hairpin structure of miRNA precursors, unannotated small RNA sequences mapped to genome were downloaded with flanking sequences and analyzed for secondary structure, Dicer cleavage sites and minimum free energy (mfe). After analysis, 141,635 reads that contained 135 novel miRNA precursors with >30 reads were identified. There were 62 novel miRNA precursors (66 mature miRNAs) shared between the three rESC lines (Table S2 in Additional file 1) and 19 (23 mature miRNAs) were homologous to reference miRNAs (miRBase V16.0) in other species (Table 1). Of the 19 miRNA precursors with homologs, 16 precursors were only conserved in humans and other primates, but not rodents.

**Table 1:** Novel miRNAs identified in rESCs

Mature miRNA sequence	MiRNA precursor location	Name	Reads		
			**IVF1.2**	**IVF3.2**	**IVF3.3**
TATGGAGGTCTCTGTCTGGCT	chr1:205,580,537-205,580,616:-	miR-1843-5p	124	159	152
TCTGATCGTTCCCCTCCATACA	chr1:205,580,537-205,580,616:-	miR-1843-3p	127	174	193
TGATGGGTGAATTTGTAGAAGG	chr1:70,976,129-70,976,208:-	miR-1262-3p	2,557	2,267	2,150
GTTGGGACAAGAGAACGGTCTT	chr1:158,332,926-158,333,000:-	miR-3122-5p	252	371	214
AATTCCCTTATGGATAATCTGG	chr2:80519804-80519882:+	miR-3938-3p	138	227	104
CATGCTAGAACAGAAAGAATGGG	chr3:106,443,245-106,443,327:+	miR-3146-3p	37	120	61
TATTTTGAGTGTTTGGAATTGA	chr4:125,407,861-125,407,939:+	miR-3145-3p	35	36	31
GAAAATGATGAGTAGTGACTGATG	chr4:128869861-128869951:+	miR-3622-3p	72	243	73
AAGAGCTTTTGGGAATTCAGGTAG	chr5:144,708,317-144,708,405:-	miR-3140-3p	151	358	200
ATATACAGGGGGAGACTCTTAT	chr7:164,327,476-164,327,555:+ chr7:164,328,696-164,328,775:+	miR-1185-3p	52	176	105
CGGGAACGTCGAGACTGGAGC	chr7:164,844,819-164,844,896:-	miR-1247-3p	127	131	32
TTAGGGCCCTGGCTCCATCTCC	chr9:73,887,081-73,887,164:+	miR-1296-5p	33	123	53
TCGACCGGACCTCGACCGGCTCG	chr9:103,084,702-103,084,783:-	miR-1307-5p	291	872	944
ACTCGGCGTGGCGTCGGTCGTGG	chr9:103,084,702-103,084,783:-	miR-1307-3p	1,936	3,975	3,030
GACTCTAGCTGCCAAAGGCGCT	chr11:98,713,141-98,713,223:+	miR-1251-5p	40	97	59
TGTGGGACCTCTGGCCTTGGC	chr11:105706113-105706195:+	miR-3922-3p	192	250	248
TGCGGGGCTAGGGCTAACAGCA	chr16:11,829,804-11,829,901:+	miR-744-5p	11,447	24,797	14,171
CTGTTGCCACTAACCTCAACC	chr16:11,829,804-11,829,901:+	miR-744-3p	75	81	104
TTTCCGGCTCGCGTGGGTGTGT	chr16:18,891,345-18,891,423:-	miR-1180-3p	30	133	77
CCGTCCTAAGGTTGTTGAGTT	chrX:68,990,098-68,990,174:+	miR-676-3p	46	206	206
TTCATTCGGCTGTCCAGATGTA	chrX:113,236,410-113,236,505:+	miR-1298-5p	246	893	852
CATCTGGGCAACTGACTGAACT	chrX:113,236,410-113,236,505:+	miR-1298-3p	30	98	58
TGAGTACCGCCATGTCTGTTGGG	chrX:113,271,154-113,271,232:+	miR-1911-5p	88	843	473

### MiRNA target gene prediction and functional classification

Three programs were used to predict miRNA target genes. Using the TargetScan program, a custom set of Perl codes were downloaded from the TargetScan database (Release 5.1, http://www.targetscan.org/). Conserved and non-conserved predicted patterns of miRNA targets were considered separately. For PITA and miRanda, we used 238 expressed miRNAs (number of reads ≥30) and rhesus macaque 3'UTRs orthologous to annotated human 3'UTRs as inputs to predict miRNA targets. Over-representation of predicted miRNA targets for both conserved and non-conserved patterns based on the TargetScan program in the Kyoto Encyclopedia of Genes and Genomes (KEGG) pathway is shown in Table S3 in Additional file 1 (P≤0.05, after Benjamini Hochberg correction). Gene enrichment patterns obtained by various methods were similar to each other, indicating that miRNAs correlated to functionally critical roles in rESCs. For example, predicted targets involved in the MAPK (hsa04010), Wnt (hsa04310) and TGF-beta signaling pathways (hsa04350), and pathways involved in cancer (hsa05200) were significantly enriched (Table S3 in Additional file 1).

### MiRNA-target interaction network analysis

**Figure 4:**
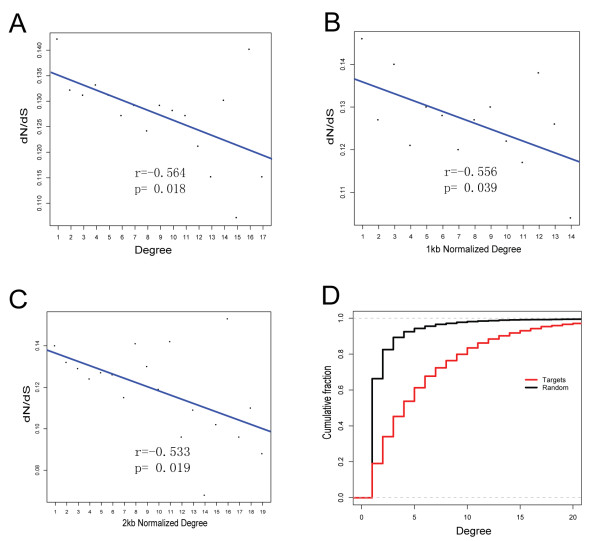
**The degree of genes regulated by miRNAs is negatively correlated with their evolutionary rate in rESCs**. (A): The correlation coefficient (r = -0.564 with *P *= 0.018 based on Pearson's correlation) between dN/dS ratios and degrees of genes regulated by miRNAs in rESCs; (B): The correlation coefficient (r = -0.556 with *P *= 0.039 based on Pearson's correlation) between dN/dS ratios and degrees with normalization of 3'UTR length per 1 kb; (C): The correlation coefficient (r = -0.533 with *P *= 0.019 based on Pearson's correlation) between dN/dS ratios and degrees with normalization of 3'UTR length per 2 kb; (D): The degree of miRNAs and their targets in the random network is significantly lower (*p *< 2.2e^-16 ^with a Wilcoxon rank sum test) compared with those in the MT network.

A systematic survey of miRNAs that interacted with targets may provide a better understanding of miRNA regulation in stem cells. Thus, we analyzed potential interactions between miRNAs and their targets, which is termed as the miRNA-target network (MT network). All miRNAs and their targets were used to generate a bipartite graph of miRNA-target interactions.. Nodes represent miRNAs or target genes and edges correspond to interactions between miRNAs with target genes. Node degree in the MT network is the number of connections or edges with other nodes. For each target gene node, degrees represent the number of miRNAs targeting the gene. Globally, the MT network was comprised of 8,934 nodes with 48,546 edges. Using MT network degree analysis (see methods), the degree of genes regulated by miRNAs was negatively correlated (r = -0.564 with p = 0.018 based on Pearson's correlation) with their evolutionary rate (dN/dS) (Figure 4A). Similarly, a previous study reported a negative correlation between the degree and evolutionary rate of proteins in a protein-protein interaction network [27].

To exclude factors with potential effects on the observed negative correlation, we tested the effect of 3'UTR length. After length normalization, the negative correlation remained (Figure 4B,4C). Another factor was a potential ascertainment bias caused by miRNA target prediction and identification of orthologous macaque 3'UTRs. To evaluate this possibly, we randomized the scale-free MT network based on Barabasi-Albert model. The degree of miRNA and target genes in the random network was significantly lower (p < 2.2e^-16 ^with a Wilcoxon rank sum test) compared with that observed in the MT network (Figure 4D). A significant correlation was not observed in the random network, which further supported the negative correlation in the MT network.

### Comparison of miRNA profiles from mouse, human and rhesus macaque

To compare ESC miRNA profiles between species, we retrieved previously reported mouse and human ESC miRNA profiles. To avoid a cross-talking bias due to various sequencing techniques and data sizes, we selected Solexa sequencing datasets from mouse and human ESCs [14,28]. MiRNA expression frequencies were normalized to each other by determining the expected frequency in mapped reads per million. MiRNA expression levels were calculated as the mean of three samples. The three species expressed 138 common miRNAs. Clustering analysis based on the 138 shared miRNAs revealed that miRNA expression profiles were more similar between human and rhesus macaque (Fig. S3 in Additional file 1), which was consistent with their evolutionary relationship. A number of miRNAs were stably and highly expressed between species, such as the miR-17 family, miR-21 and miR-103, which suggested conserved functions in ESCs during evolution.

Of the 138 common miRNAs the 'stemness' miR-290-295 cluster [13,29] was the highest expressed in mouse ESCs. However, primate homologs the miR-290-295 and miR-371-373 clusters were not highly expressed in human and rESCs. Instead, the miR-302 cluster was the highest expressed in human and rhesus macaque [14,30,31], indicating the functional divergence of stemness miRNA clusters in primate lineages. The miR-290-295 cluster contains multiple mature miRNAs with seed sequences similar or identical to those in the miR-302 cluster [6]. MiR-290-295 and miR-302 clusters are transcriptionally regulated by Oct-4/Sox2 [29]. Taken together, these observations suggest that these two clusters are similarly expressed, regulate common target genes and are functionally conserved in ESCs derived from various species.

**Figure 5:**
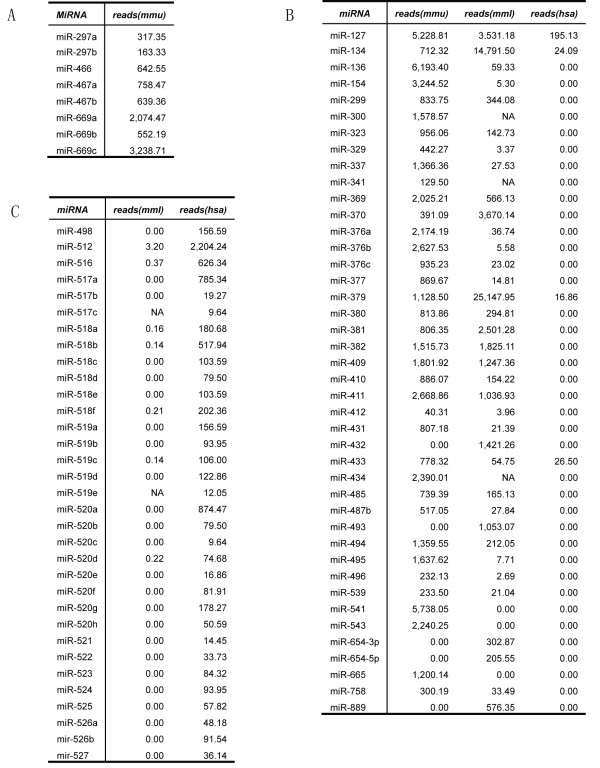
**Expression differences of three miRNA clusters among human, rhesus macaque and mouse ESCs**. (A): MiR-467cluster expression in mouse (mmu) ESCs; (B): Expression of the miRNA cluster in the imprinted Dlk1-Dio3 region in mouse, rhesus macaque (mml) and human (hsa) ESCs; (C): C19MC expression in rhesus macaque (mml) and human (hsa) ESCs. Reads were normalized to the mapped reads per million.

There are three miRNA clusters differentially expressed in mouse, human and rESCs. The chromosome 19 miRNA cluster (C19MC), a primate-specific miRNA cluster [32], was enriched in human ESCs, but almost absent in rESCs (Figure 5C). A conserved miRNA cluster within the imprinted Dlk1-Dio3 region was highly expressed in mouse and rhesus macaque ESCs, but rarely expressed in human ESCs (Figure 5B), which was consistent with results from four other human ESC lines [31].The miR-467 cluster in the *sfmbt2 *gene intron region was only expressed in mouse ESCs (Figure 5A), embryos and newborn ovaries [33,34], but not in human and rhesus macaque ESCs, consistent with previous reports [29].

## Discussion

We established three rESC lines (IVF1.2, IVF3.2 and IVF3.3) and profiled miRNA expression using Solexa sequencing. MiRNA expression profiles were generally conserved between the three rESC lines, sharing 92.4% of expressed miRNAs. IVF3.2 and IVF3.3 were more similar because of derivation from identical sperm and ovum donors. Similarly, Navara *et al*. [35] reported that pedigreed rESCs express homogeneous gene profiles. Compared with known miRNAs in our data set, most novel miRNAs were moderately expressed. Novel miRNA biological functions and association with stemness are yet to be elucidated.

We predicted miRNA targets in rESCs using three independent algorithms. Targets involved in TGF-β, MAPK and Wnt signaling pathways were significantly enriched. These pathways play important roles in mouse and human ESC differentiation [36-38]. This observation suggests that in addition to transcriptional level regulation by stemness-associated factors such as Oct4 and Nanog, miRNAs may also play an important role in maintaining ESC self-renewal and pluripotency at the post-transcriptional level. Moreover, over-represented miRNA targets involved in cancer pathways support the observation that cancer and ESCs share pathways for self-renewal and proliferation [38].

Our systematic investigation of the MT network suggested that the degree of genes regulated by miRNAs was negatively correlated with their evolutionary rate in rESCs. Genes with more miRNA regulators evolve more slowly. This was anticipated in accordance to a rule of "the more conserved, the deeper regulated" has been proposed in protein-protein interaction networks, in which hubs or central proteins are functionally essential and usually lethal after gene knockout [27]. Therefore in the MT network, hub targets should be more regulated due to selective pressure, which indicates the fine regulation of miRNAs in ESCs.

The cross-species comparison indicated that the majority of shared miRNAs were stably expressed between species and consistent with miRNA sequence-level conservation. This observation suggested that miRNA expression is under stabilizing selection due to functional constraints on miRNAs in ESCs. Despite this close relationship, miRNA expression differences existed between human and rhesus macaque ESCs. The chromosome 19 miRNA cluster (C19MC) is a primate-specific miRNA cluster and contains >30 mature miRNAs. These clustered miRNAs are expressed in human ESCs, placenta and fetal brain [14,31,32,39], suggesting that they are involved in embryogenesis. However, the C19MC cluster is almost absent in rESCs. This expression difference suggests that even between closely related species, there may be a significant miRNA expression divergence and regulatory differences during embryogenesis between rhesus macaques and humans. In addition, the miRNA cluster in the imprinted Dlk1-Dio3 region was conserved at the sequence level between mice, rhesus macaques and humans. Liu *et al *reported that the expression of this cluster is positively correlated to mouse stem cell pluripotencies (ESCs vs iPS cells) [40]. We found that this cluster was enriched in mouse and rhesus macaque ESCs, but rare in human ESCs. Recently, it was reported that diverse temporal origins may be responsible for observed differences between mouse and human ESCs [41]. Our data suggests that miRNA expression patterns may provide a new strategy for investigating interspecies differences in ESC pluripotency.

## Conclusions

Our results indicate that miRNA profiles are highly conserved and consistent between the three rESC lines, and these miRNAs may play critical roles in the maintenance of self-renewal and pluripotency in rESCs, particularly in differentiation pathways. Further analysis of the miRNA-target network indicates that the degree of genes regulated by miRNAs is negatively correlated with their evolutionary rate in rESCs. This observation suggests important roles of miRNAs in post-transcriptional regulation. Moreover, cross-species comparison of ESCs revealed an overall conservation of miRNA expression patterns, indicating stabilizing selection on miRNA expression in ESCs during evolution. However, we identified three miRNA clusters (miR-467, the miRNA cluster in the imprinted Dlk1-Dio3 region and C19MC) that show distinct differences between mouse, human and rhesus macaque ESCs, which suggests species and/or lineage specific miRNA regulatory changes during evolution.

## Methods

### rESC isolation and characterization

rESCs were derived from the ICM of rhesus macaque blastocyst-stage embryos and characterized using standard protocols. rESC isolation and characterization details are provided in the supplemental Methods in Additional file 1.

### RNA extraction and small RNA sequencing

Total RNA was extracted using a mirVana miRNA isolation kit (Ambion). According to the manufacturer's instructions (Illumina), the small RNA fraction between 18-30 nt was isolated from total RNA by PAGE (polyarclymide gel electrophoresis) purification and ligated to a pair of adaptors at the 5' and 3' ends. Small RNA molecules were converted to cDNA and amplified by RT-PCR using adaptor primers. Unique 6 bp indexed sequences were added to 5' adapters for multiplex sequencing. Purified DNA was directly used for cluster generation and sequencing analysis using an Illumina Genome Analyzer. These data have been deposited in the GEO database (GSE27886).

### Small RNA annotation and novel miRNA prediction

For clean reads, the original 35 nt reads from sequencing were filtered by trimming the 5' and 3' adaptors, eliminating contaminants, and inadequate (<18 nt) and low quality reads. For detecting known miRNAs, clean reads were matched to reference miRNA precursor sequences in the rhesus macaque (miRBase version 16.0) using Blastall (-p blastn -F F -e 0.01). MiRNA absolute expression was the sum of clean reads mapped to the precursor. For annotating degradation tags of rRNA, tRNA,small cytoplasmic RNA (scRNA), small nuclear RNA (snRNA) and small nucleolar RNA (snoRNA), clean reads were aligned with Genbank and Rfam 9.1 databases using Blastall. For identifying repeat associated RNA and degradation tags of mRNA, clean reads were overlapped with repeat sequences, exons and introns of mRNAs. In the initial alignment and annotation, some small RNA tags were mapped to more than one category. For uniquely mapped reads, the following priority rules were applied: rRNA/tRNA/scRNA/snRNA/snoRNA (Genbank > Rfam) > known miRNA > repeat > exon > intron. Unannotated small RNA tags were termed as 'unann'. Mireap (http://sourceforge.net/projects/mireap/) was used to predict novel miRNAs.

### MiRNA expression profiling analysis

Based on the randomly selected 10,000 bins for each chromosome with each bin spanning a 50 bp window (210,000 bins in 21 chromosomes), miRNAs with sporadic reads were filtered out and miRNAs with >30 reads (p < 0.0001 based on Poisson distribution simulation) were treated as true reads for further analyses. miRNA expression profiles were compared and evaluated in rECSs at cluster and individual miRNA levels. A miRNA cluster was defined as consecutive miRNAs with a ≤10 kb inter-miRNA distance (MID). For each level, two independent methods were used for confirmation. Var and C.V. were used to survey miRNA member divergences in a cluster. Var was used to represent a divergence measure based on Shannon entropy [42]. D.I. and Kappa Statistical analysis (*κ*) were used to compare total expressed miRNAs in rESCs. D.I. was used to evaluate relative deviation of individual miRNA expression levels in rESCs. Kappa statistical analysis was used to measure the repeatability of total miRNAs expression between any two samples (supplemental methods in Additional file 1).

### MiRNA target prediction

To predict miRNA target genes, TargetScanS [43], miRanda (http://www.microrna.org/microrna/home.do) and PITA (http://genie.weizmann.ac.il/pubs/mir07/mir07_prediction.html) were used. Common miRNAs (≥30 reads) were used to predict target genes. For each miRNA, the reference mature miRNA sequence in miRBase was used. For 3'UTR identification in the rhesus macaque, human annotated 3'UTRs from Refseq and orthologous 3'UTRs from six species (human, chimpanzee, macaque, mouse, rat and dog) were extracted from multi17way aligned files downloaded from the UCSC database [44]. Orthologous 3'UTRs were aligned using the MUSCLE [45] program to obtain multi6way 3'UTR files required for analysis.

Gene ontology (GO) analyses of predicted miRNA targets were performed using t DAVID Bioinformatics Resources [46] (http://david.abcc.ncifcrf.gov/home.jsp). Ascertainment biases such as a relatively low number of annotated miRNAs, 3'UTRs in the rhesus macaque, ≥30 cutoffs for miRNA reads and false predicted miRNA targets were assessed by simulating the bias with 240 randomly synthesized *in silico *22 nt miRNAs (equal number with true expression).

Potential correlations between miRNA (or target) degrees connected in the MT network, miRNA expression and target evolution were evaluated (supplemental methods in Additional file 1).

## Abbreviations

ESCs: embryonic stem cells; ICM: inner cell mass; EB: embryoid body; miRNA: microRNA; nt: nucleotide; scRNA: small cytoplasmic RNA; snRNA: small nuclear RNA; snoRNA: small nucleolar RNA; Var: variability; C.V.: coefficient of variation; D.I.: differential index; *κ*: Kappa Statistic; MT network: miRNA-target regulatory network; iPS cell: induced pluripotent stem cell; C19MC: chromosome 19 miRNA cluster; mfe: minimum free energy; SOAP: Short oligonucleotide alignment program; SCID: severe combined immune deficiency; mEFs: mouse embryonic fibroblasts; dN/dS: nonsynonymous vs. synonymous substitution rate.

## Authors' contributions

ZS and QW: data collection, assembly, analysis and interpretation, and manuscript writing; YZ: data analysis and interpretation, and manuscript writing; XH: provision of study material; WJ and BS: conception and design, data interpretation, financial support, manuscript writing and final approval of manuscript. All authors read and approved the final manuscript.

## Supplementary Material

Additional data file 1 is a DOC file with Supplemental Methods, Figures (S1, S2, S3, S4) and Tables (S1, S2, S3, S4). Click here for file
