# Supplementary material for: MicroRNA profiling of rhesus macaque embryonic stem cells
Source: BMC Genomics. 2011 May 31;12:276. doi: 10.1186/1471-2164-12-276 (PMC3117859; doi:10.1186/1471-2164-12-276)
Supplement: Additional data file 1 — is a DOC file with Supplemental Methods, Figures (S1, S2, S3, S4) and Tables (S1, S2, S3, S4). [file 1471-2164-12-276-S1.DOC]

**Additional Data**

**Supplemental materials and methods**

**Animals**

Adult rhesus macaques were supplied by Kunming Primate Research Center, and housed in individual cages when they were used for the present study. All animal procedures were approved by the Institutional Animal Care and Use Committee of the Kunming Primate Research Center, the Chinese Academy of Sciences.

**Ovarian Stimulation, recovery of oocytes, fertilized *in vitro*, and embryo culture**

Ovarian stimulation and recovery of oocytes was conducted as described previously[1]. Briefly, cycling females were injected to follicular stimulation using twice-daily intramuscular injections

of 18 IU of recombinant human FSH (rhFSH) (Gonal FTM, Laboratories Serono SA, Aubonne, Switzerland)for 8 days, with 2000 IU of hCG (Lizhu Groups, Shenzhen, China) injected on day 9.

Cumulus-oocyte complexes were collected from animals anaesthetized with ketamine (10–12 mg/kg) by laparoscopic follicular aspiration, 30–34 h following hCG administration. Follicular contents were placed in HEPES-buffered Tyrode’s albumin lactate pyruvate medium (TALP) containing 0.3% bovine serum albumin(BSA) at 37℃. Oocytes were stripped off cumulus cells by pipetting after transitory exposure (<1 min) to hyaluronidase (0.5 mg/ml).

Mature oocytes were fertilized in vitro with capacitated and stimulated sperm (2×10^7^ /ml) diluted in Tyrode albumin lactate pyruvate culture medium, according to Bavister et al. Fertilized oocytes were then cultured in hamster embryo culture medium 9 (HECM-9) containing 10% fetal bovine serum (FBS; HyClone, Logan, UT, http://www.hyclone.com) at 37℃ with 5% CO_2_.

**Embryonic stem cells derivation and culture**

Nature hatched or full expanded blastocysts with distinct inner cell masses (ICMs) were selected to derive embryonic stem cells. Zonae pellucidae were removed by transitory exposure to 0.5% pronase, and ICMs were isolated using immunosurgery[2]. Isolated ICMs were plated on Nunc four-well dishes containing mitotically inactivated mouse embryonic fibroblasts (mEFs) and cultured in KO-DMEM medium containing 10% FBS, 10% KO-SR supplemented with 10ng/ml bFGF(Chemicon), 10 ng/ml Activin A(R＆D), 1000 IU/ml human recombinant LIF(Chemicon), 1% nonessential amino acids (Invitrogen), 0.1mM β-mercaptoethanol, 1% penicillin-streptomycin-l-glutamate(PSG)(Invitrogen). ICMs were attached to the mEFs and initiated outgrowth were manually dissociated into small cell clumps with a microscalpel and replated onto new mEFs. After the first passage, colonies with ESC-like morphology were selected for further propagation, characterization and freezing. When all colonies were uniform ESC-like morphology, culture medium was replaced by KO-DMEM medium containing 20% KO-SR supplemented with 5ng/ml bFGF(Chemicon), 1% nonessential amino acids, 0.1mM β-mercaptoethanol, 1% PSG. The medium was changed daily, and ESC colonies were split every

4-6 days manually or by disaggregation in collagenase IV (1 mg/ml, at 37°C for 15minutes; Invitrogen) and replating onto dishes with fresh feeder layers. Cultures were maintained at 37°C, with 5% CO_2_.

**Mycoplasma test**

we conducted mycoplasma test useing Venor®GeM-Mycoplasma Detection Kit (Cat.No.11-1050; Minerva Biolabs; Germany) during ESC culture~~s~~ to make sure there was no mycoplasma contamination in all three samples (Fig S4 in Additional file 1).

**Differentiation of ESCs *in vitro***

For embryoid body (EB) formation, entire ESCs colonies were detached from feeder cells by exposure to collagenase IV (1 mg/ml, at 37°C for 15minutes) and transferred into agar-coated dishes and cultured in suspension in Dulbecco.s modified Eagle.s medium (DMEM, high glucose, without sodium pyruvate; Gibco)containing 15% FBS, 1% nonessential amino acids, 0.1mM β-mercaptoethanol, 1% PSG. After 5-7days, EBs were transferred into gelatin-coated dishes allowing attachment for further differentiation. Medium was changed every other day.

**Teratoma formation**

Entire ESCs colonies were detached from feeder cells by exposure to collagenase IV (1 mg/ml, at 37°C for 15minutes) and about 2 million undifferentiated ESCs from each cell line were harvested and injected into the hind leg muscle of 4-week-old SCID male mice using an 18-gauge needle. 6-7 weeks after injection, mice were sacrificed, and teratomas were dissected, sectioned, and histologically characterized for the presence of representative tissues of all three germ layers.

**Immunohistochemical staining**

Cells were fixed with 4% paraformaldehyde in PBS for 20 minutes at room temperature, and

permeabilized with 0.1%TritonX-100 in PBS for 10 minutes at room temperature. After blocked with 3% BSA, cells were stained with primary antibodies. Cells were then rinsed three times with PBS and incubated for 60 minutes with fluorescein isothiocyanate (FITC)–conjugated secondary antibody (Santa Cruz Biotechnology). Negative controls for each fluorophore-conjugated secondary antibody were carried out without the primary antibody, and nonspecific binding of secondary antibodies was detected.

**Reverse transcription-polymerase chain reaction**

Total RNA was extracted using a TRIZOL RNA isolation kit (Invitrogen Corporation) according to the manufacturer’s instructions. Potential contamination from genomic DNA was eliminated by DNase digestion. Cytoplasmic RNA was reverse-transcribed to single-stranded cDNA. Aliquots of cDNA were used as a template for polymerase chain reaction (PCR) amplification with individual primer pairs for specific genes. The sense and antisense primer sequences, corresponding PCR condition, and product sizes were shown in Table 1. 5µl of PCR products were separated on a 1.5% agarose gel and visualized by ethidium bromide staining. the primers and the RT-PCR parameters were shown in Table S4 of Additional file 1..

**Cytogenetic analysis**

Mitotically active PESCs in log phase were incubated with 120 ng/ml colcemid for 90 minutes at 37°C in 5% CO_2_. Entire ESCs colonies were detached from feeder cells by exposure to collagenase IV (1 mg/ml, at 37°C for 15minutes) and treated with 0.05% trypsin at 37°C for 2 minutes and centrifuged at 200*g* for 5 minutes. The cell pellet was gently resuspended in 0.075 M KCl and incubated for 20 minutes at 37°C followed by fixation with methanol/glacial acetic acid (3:1). Fixed cells were dropped on wet slides, air dried, and baked at 90°C for 1 hour. G-banding was performed as described previously.

**Alkaline phosphatase staining**

Cocultures were fixed with 4% paraformaldehyde in PBS for 15 minutes. After a washed with PBS, Alkaline phosphatase staining was performed using a kit containing BCIP/NBT (5-bromo-4-chloro-3-indolyl phosphate/Nitro Blue Tetrazolium) as substrate.

**Flow cytometry analysis of purity**

For purity analysis, harvested rESCs colonies were dissociated into single cell by 0.05% typsin with EDTA. Cells were fixed with 4% paraformaldehyde and permeabilized with 0.1% Triton X-100. After blocked with 1% BSA in PBS, cells were stained with Oct-3/4 primary antibody(Santa Cruz) or isotype antibody(Santa Cruz) for 40 minutes at 37℃. Then cells were rinsed three times with PBS and incubated for 30 minutes with the corresponding FITC -conjugated Goat anti-Mouse secondary antibody (Santa Cruz). At last, stained cells from each sample were analyzed by Flow Cytometry (BD).

**MiRNA expression profiling analysis**

Here, we adopted four independent approaches to compare and evaluate miRNA expression profiles among three samples. The former two methods, variability (*Var*,Ⅰ) and coefficient of variation (*C.V.*, Ⅱ), were employed to survey the expression of miRNA clusters in each sample. The latter two methods, differential index (*D.I.*, Ⅲ) and Kappa Statistic ($\kappa$,Ⅳ-Ⅸ), were applied to compare total expressed miRNAs among three samples.

$Var=-\sum_{i=1}^{n} \frac{n_{i}}{N}\ln\frac{n_{i}}{N}$, (Ⅰ)

where $n_{i}$represents the normalized number of miRNA reads, $n$(>=3) is the total number of miRNAs in a cluster and *N* is the maximum number of miRNA reads (*max*{$n_{i}$}) in a cluster.

$CV=100\%\times\frac{s}{\overline{x}}$, (Ⅱ)

where *s* represents the standard deviation of normalized miRNA reads in a cluster and $\overline{x}$ is the mean value of normalized miRNA reads in a cluster.

*D.I*. = $\frac{x_{i}}{\max(x_{i})},i\in1,2,3$, (Ⅲ)

where $x_{i}$ represents the normalized miRNA expression in the sample *i*.

|  |  | Sample1 | |  |
| --- | --- | --- | --- | --- |
| Sample2 |  | $\geq$30 reads | <30 reads |  |
|  | $\geq$30 reads | k | l | a1=k+l |
|  | <30 reads | m | n | a2=m+n |
|  |  | b1=k+m | b2=l+n | S |

(Ⅳ)

$p_{o}=\frac{(k+n)}{S}$ (Ⅴ)

$p_{e}=\sum a_{i}b_{i}$ (ⅤI)

$\kappa=\frac{(p_{o}-p_{e})}{(1-p_{e})}$, (Ⅶ)

$z=\frac{k}{se(k)}$, (Ⅷ)

$se(k)=\sqrt{\frac{1}{n(1-p_{e})^{2}}\times\left\{\left.p_{e}+p_{e}^{2}-\sum_{i=1}^{c} [a_{i}b_{i}(a_{i}+b_{i})]\right\}\right.}$, (Ⅸ)

where k, l, m , n represent the number of miRNA $\geq$ or < 30 reads. S represents the sum of miRNAs for two samples. $p_{o}$ represents the observed consistent probability between two samples, and $p_{e}$ for the expected consistent probability. $z$ is a statistic to test the kappa value.

**MiRNA-target regulatory network**

MT Network is visualized and topologically analyzed in the Cytoscape[3-4] and plug-in (<http://med.bioinf.mpi-inf.mpg.de/netanalyzer/index.php>). For evolutionary analysis of miRNA targets, we calculated *Ka/Ks* ratio for those targets between human and macaque, using mouse as outgroup implemented in codeml of PAML[5] with M0 model, NSsite=0, and F3x4 parameters. We next calculated the correlation of degree of miRNA targets with evolutionary rate and further evaluate the correlation based on a *z*-score as:

$z=\frac{r-\overline{r}_{rand}}{\sigma_{rand}}$, where $r$ is the observed linear correlation coefficient, $\overline{r}_{rand}$ is the randomized linear correlation coefficient based on 1000 bootstrappings, and the$\sigma_{rand}$ is the standard deviation of $\overline{r}_{rand}$. The p-value is then calculated from$p=erfc(\left|z\right|)/\sqrt{2}$, where erfc is the complement error function calculated from$erfc(x)=1-erf(x)=\frac{2}{\sqrt{\pi}}\int_{x}^{\infty} e^{-t^{2}}dt$. What’s more, we used Random Networks plug-in implemented in the Cytoscape to construct a scale-free randomized MT network based on Barabasi-Albert model to compare with the true regulatory network.

**Reference:**

1. Yang J, Yang S, Beaujean N, Niu Y, He X, Xie Y, Tang X, Wang L, Zhou Q, Ji W: **Epigenetic marks in cloned rhesus monkey embryos: comparison with counterparts produced in vitro.** *Biology of reproduction* 2007, **76:**36.

2. Mitalipov S, Kuo H, Byrne J, Clepper L, Meisner L, Johnson J, Zeier R, Wolf D: **Isolation and characterization of novel rhesus monkey embryonic stem cell lines.** *Stem cells* 2006, **24:**2177-2186.

3. Shannon P, Markiel A, Ozier O, Baliga NS, Wang JT, Ramage D, Amin N, Schwikowski B, Ideker T: **Cytoscape: a software environment for integrated models of biomolecular interaction networks.** *Genome Research* 2003, **13:**2498.

4. Zhang Y, Guan DG, Yang JH, Shao P, Zhou H, Qu LH: **ncRNAimprint: a comprehensive database of mammalian imprinted noncoding RNAs.** *Rna* 2010, **16:**1889.

5. Yang Z: **PAML 4: phylogenetic analysis by maximum likelihood.** *Molecular biology and evolution* 2007, **24:**1586.

**Supplementary figure legends**

**Figure S1. Karyotype analysis of the three embryonic stem cell lines.**

**Figure S2. Purity analysis of the three rESC lines.** The Oct-4 expression in ESCs was detected by FACS with FITC staining (green) , isotype control (red) was used to remove the non-specific staining.

**Figure S3. Clustering analysis reveals that miRNA expression profiles are more similar between rESCsand hESCs.** Shown here are heatmap representations of miRNA expression profiles among mESCs，rESCs and hESCs based on 138 shared miRNAs. MiRNA expression frequencies were normalized to each other by determining the expected frequency in mapped reads per one million and log-transformed.

**Figure S4. The mycoplasma test for the three ESC lines is negative.** When running the PCR with internal control DNA, a successfully performed reaction is indicated by a 191 bp band on the agarose gel. Mycoplasma are specifically detected by amplifying a highly conserved rRNA operon, or more specifically, the 16S rRNA coding region in the mycoplasma genome. The generated mycoplasma DNA amplicon shows a size of approx. 267 bp.

Fig S1.


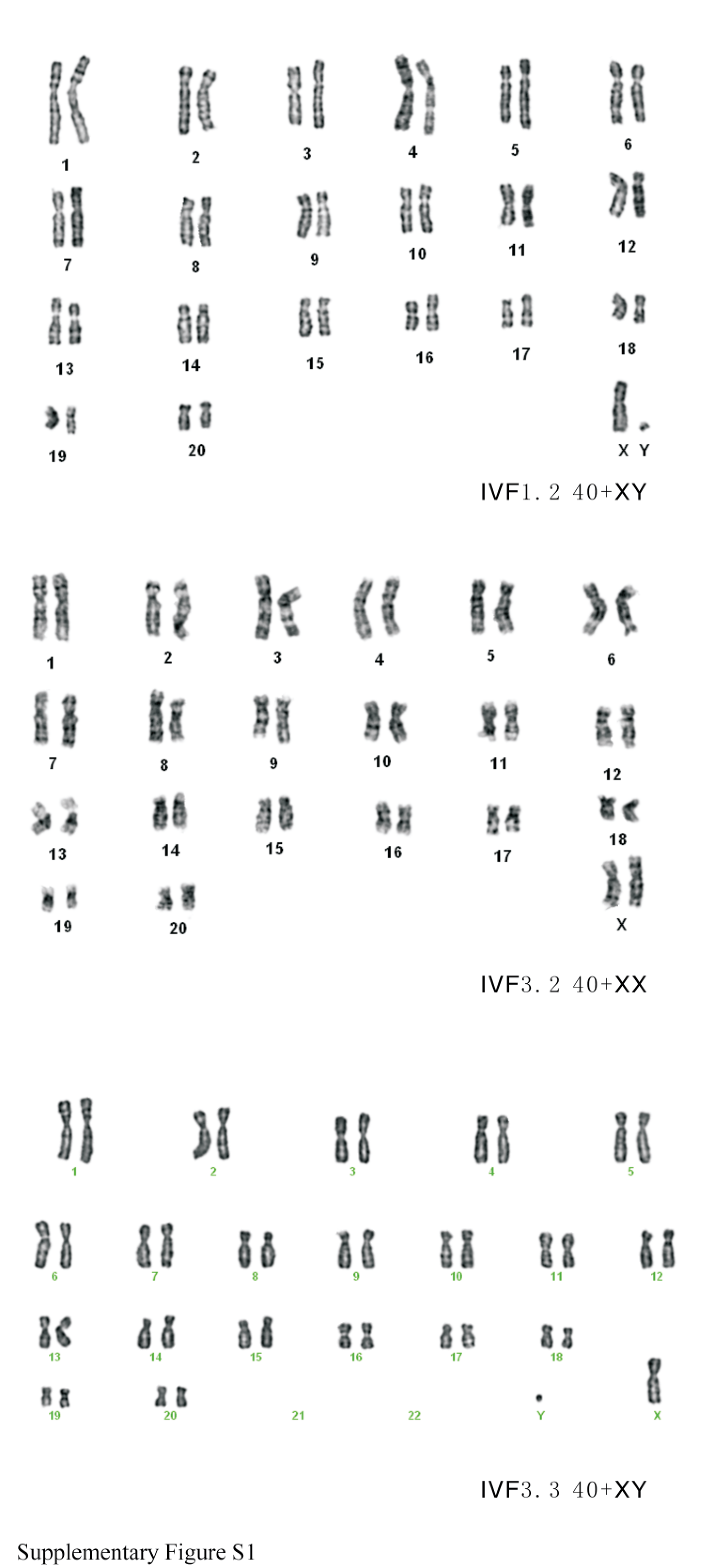


Fig S2.


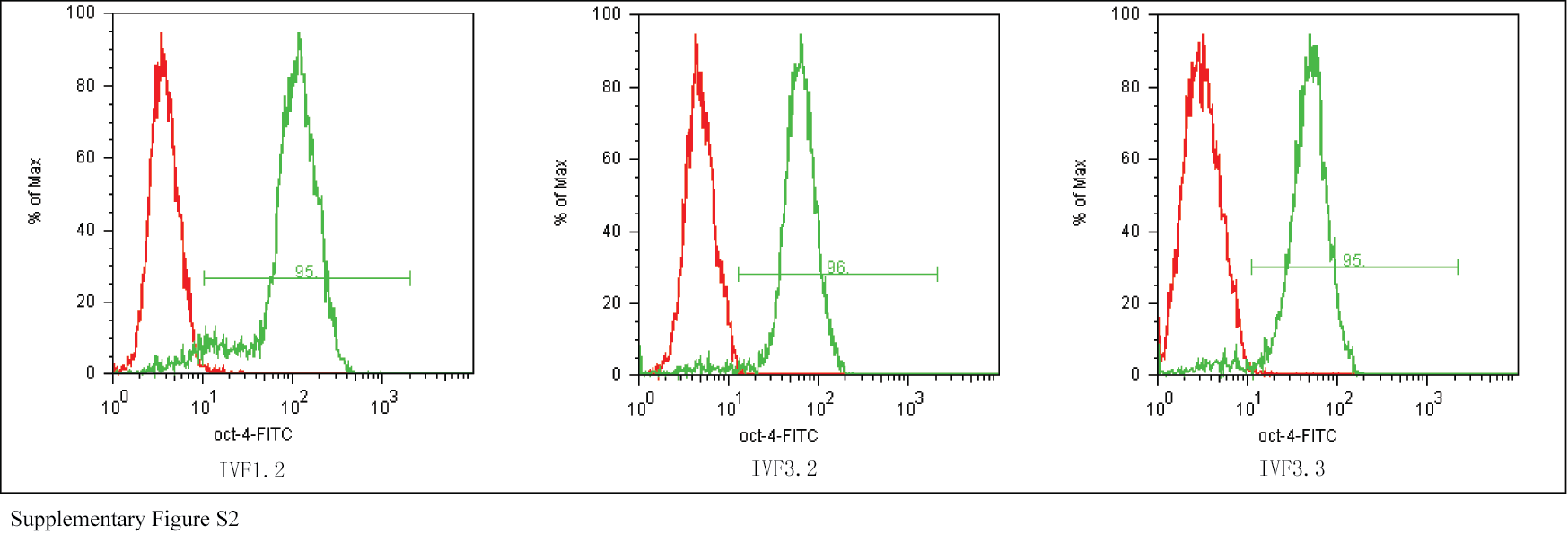


Fig S3.


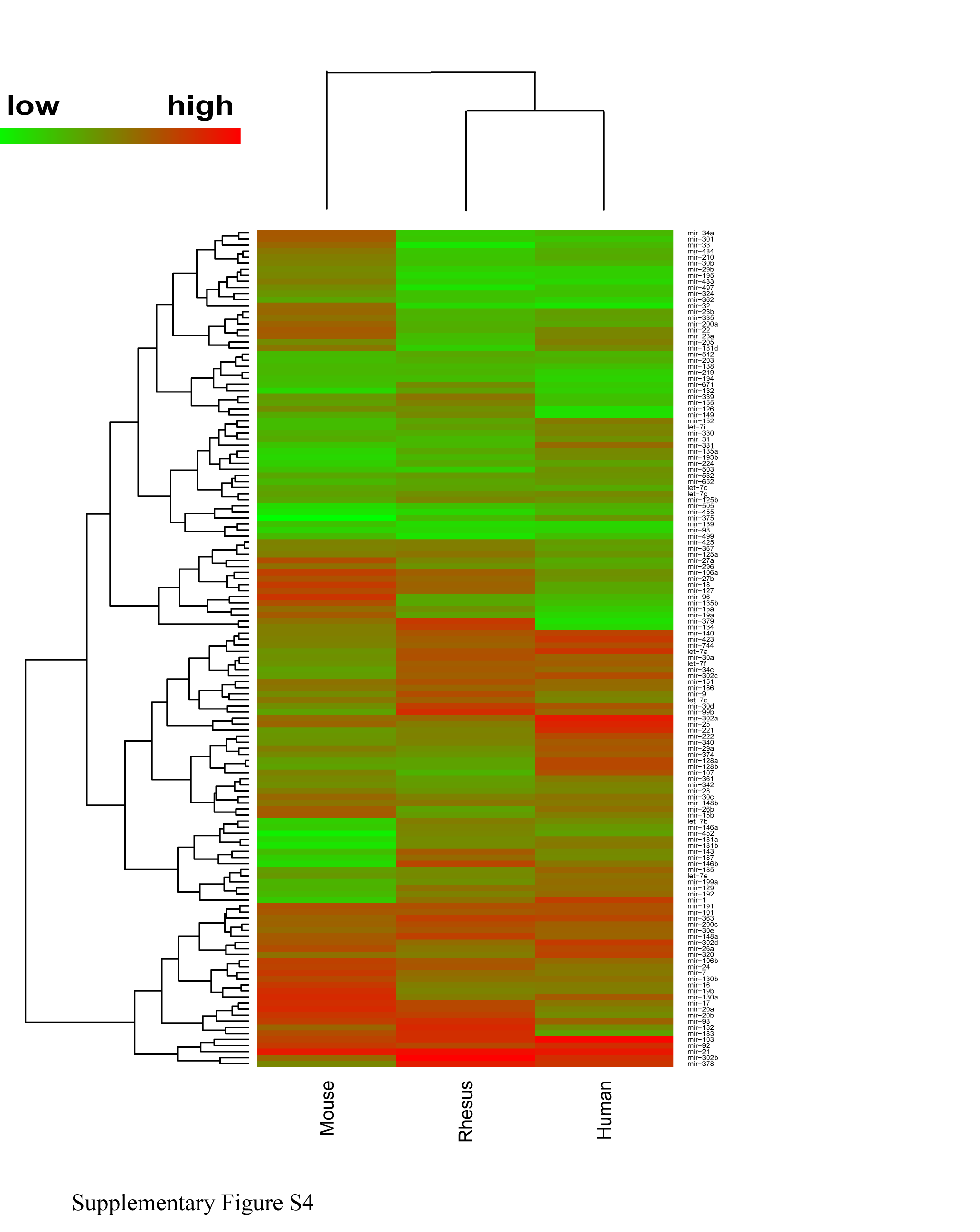


Fig S4.


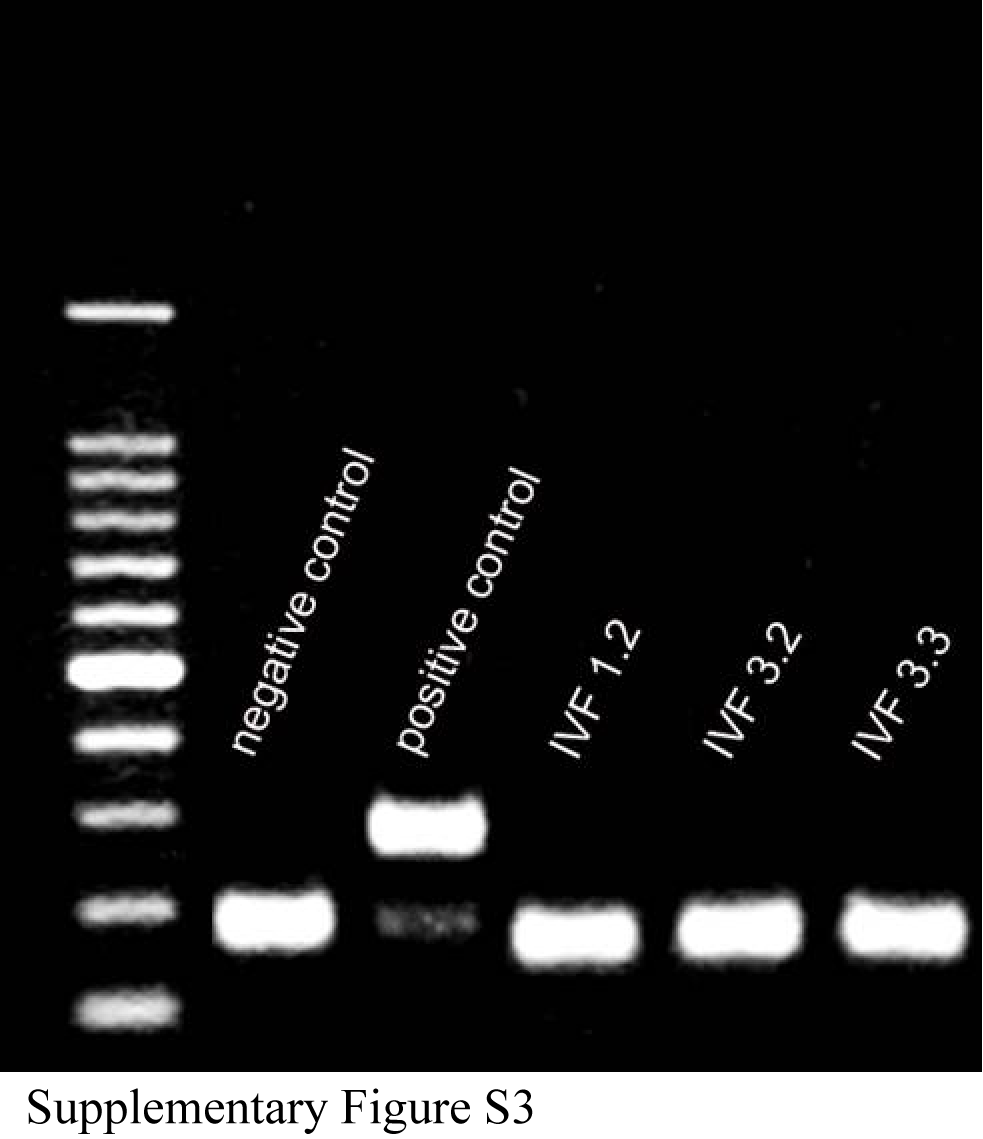


191bp

267bp

Table S1. The absolute counts of known miRNAs were detected in IVF1.2, IVF3.2 and IVF3.3.

| Name | IVF1.2 counts | IVF3.2 counts | IVF3.3 counts |
| --- | --- | --- | --- |
| mml-let-7a | 294,776 | 54,333 | 29,486 |
| mml-let-7b | 144,981 | 6,584 | 4,646 |
| mml-let-7c | 325,928 | 17,346 | 15,891 |
| mml-let-7d | 21,348 | 1,009 | 816 |
| mml-let-7e | 27,582 | 3,786 | 2,555 |
| mml-let-7f | 243,301 | 22,571 | 19,591 |
| mml-let-7g | 22,569 | 2,192 | 1,967 |
| mml-let-7i | 13,548 | 1,060 | 1,103 |
| mml-miR-1 | 12,711 | 14,537 | 7,545 |
| mml-miR-100 | 14,425 | 589 | 620 |
| mml-miR-101 | 15,204 | 19,612 | 20,000 |
| mml-miR-103 | 83,585 | 209,141 | 142,924 |
| mml-miR-105 | 1,063 | 3,077 | 2,778 |
| mml-miR-106a | 9,951 | 21,929 | 16,597 |
| mml-miR-106b | 19,073 | 27,905 | 23,328 |
| mml-miR-107 | 252 | 570 | 414 |
| mml-miR-10a | 126,946 | 3,444 | 4,427 |
| mml-miR-10b | 35,628 | 2,570 | 1,074 |
| mml-miR-1224 | 17 | 59 | 44 |
| mml-miR-1225-5p | 1 | 0 | 0 |
| mml-miR-1226 | 2 | 2 | 1 |
| mml-miR-122a | 921 | 156 | 214 |
| mml-miR-1230 | 0 | 1 | 0 |
| mml-miR-1240 | 1 | 0 | 0 |
| mml-miR-124a | 21 | 48 | 30 |
| mml-miR-125a-3p | 375 | 197 | 148 |
| mml-miR-125a-5p | 22,020 | 5,966 | 6,703 |
| mml-miR-125b | 53,091 | 2,398 | 2,895 |
| mml-miR-126 | 1,990 | 2,361 | 1,982 |
| mml-miR-127 | 28,981 | 17,585 | 13,761 |
| mml-miR-128a | 553 | 1,171 | 891 |
| mml-miR-128b | 514 | 1,082 | 830 |
| mml-miR-129-5p | 3,846 | 7,054 | 7,629 |
| mml-miR-130a | 2,905 | 6,715 | 4,826 |
| mml-miR-130b | 3,439 | 11,877 | 7,557 |
| mml-miR-132 | 2,618 | 851 | 916 |
| mml-miR-133b | 2 | 1 | 0 |
| mml-miR-133c | 14 | 7 | 15 |
| mml-miR-134 | 56,813 | 111,422 | 67,814 |
| mml-miR-135a | 439 | 1,230 | 712 |
| mml-miR-135b | 271 | 730 | 678 |
| mml-miR-136 | 689 | 190 | 189 |
| mml-miR-137 | 26 | 72 | 55 |
| mml-miR-138 | 582 | 492 | 361 |
| mml-miR-139-3p | 38 | 53 | 67 |
| mml-miR-139-5p | 21 | 25 | 16 |
| mml-miR-140-3p | 67,017 | 34,256 | 25,998 |
| mml-miR-140-5p | 2,197 | 1,984 | 1,558 |
| mml-miR-141 | 752 | 527 | 747 |
| mml-miR-142-3p | 103 | 71 | 57 |
| mml-miR-142-5p | 26 | 12 | 7 |
| mml-miR-143 | 348,237 | 25,033 | 20,627 |
| mml-miR-145 | 274 | 37 | 22 |
| mml-miR-146a | 22,809 | 4,109 | 3,333 |
| mml-miR-146b-5p | 56,129 | 54,521 | 50,388 |
| mml-miR-147b | 0 | 1 | 1 |
| mml-miR-148a | 52,070 | 87,475 | 68,723 |
| mml-miR-148b | 3,821 | 7,385 | 5,741 |
| mml-miR-149 | 2,219 | 3,373 | 2,671 |
| mml-miR-150 | 8 | 0 | 0 |
| mml-miR-151-3p | 14,772 | 41,138 | 29,589 |
| mml-miR-151-5p | 3,212 | 4,756 | 3,515 |
| mml-miR-152 | 17,750 | 718 | 739 |
| mml-miR-153 | 5 | 9 | 6 |
| mml-miR-154 | 53 | 21 | 19 |
| mml-miR-155 | 2,172 | 4,826 | 3,952 |
| mml-miR-15a | 941 | 2,317 | 1,801 |
| mml-miR-15b | 831 | 1,668 | 1,271 |
| mml-miR-16 | 3,512 | 5,568 | 4,132 |
| mml-miR-17-3p | 969 | 1,872 | 1,494 |
| mml-miR-17-5p | 26,392 | 62,690 | 44,507 |
| mml-miR-18 | 5,263 | 14,125 | 11,420 |
| mml-miR-181a | 17,471 | 3,645 | 2,468 |
| mml-miR-181a* | 283 | 25 | 10 |
| mml-miR-181b | 10,884 | 3,904 | 2,284 |
| mml-miR-181c | 211 | 14 | 18 |
| mml-miR-181d | 884 | 146 | 126 |
| mml-miR-182 | 117,942 | 292,935 | 214,639 |
| mml-miR-183 | 95,052 | 196,261 | 147,645 |
| mml-miR-184 | 4,434 | 223 | 161 |
| mml-miR-185 | 1,789 | 4,142 | 2,282 |
| mml-miR-186 | 10,148 | 13,632 | 12,992 |
| mml-miR-187 | 3,797 | 7,783 | 10,145 |
| mml-miR-188 | 52 | 24 | 46 |
| mml-miR-18b | 1,164 | 3,679 | 3,108 |
| mml-miR-190a | 223 | 516 | 640 |
| mml-miR-190b | 35 | 101 | 108 |
| mml-miR-191 | 31,224 | 46,269 | 31,423 |
| mml-miR-192 | 11,498 | 4,760 | 3,935 |
| mml-miR-193a-3p | 6 | 0 | 0 |
| mml-miR-193a-5p | 144 | 152 | 104 |
| mml-miR-193b | 147 | 388 | 364 |
| mml-miR-194 | 1,048 | 484 | 289 |
| mml-miR-195 | 174 | 132 | 83 |
| mml-miR-196a | 54,108 | 1,468 | 1,505 |
| mml-miR-196b | 6,219 | 939 | 657 |
| mml-miR-197 | 186 | 334 | 383 |
| mml-miR-199a | 10,133 | 329 | 368 |
| mml-miR-199a-3p | 55,431 | 1,407 | 1,478 |
| mml-miR-199a-5p | 10,133 | 329 | 368 |
| mml-miR-19a | 274 | 985 | 885 |
| mml-miR-19b | 923 | 4,137 | 3,299 |
| mml-miR-200a | 590 | 330 | 486 |
| mml-miR-200c | 42,660 | 28,113 | 35,017 |
| mml-miR-203 | 1,318 | 611 | 531 |
| mml-miR-204 | 71 | 168 | 240 |
| mml-miR-205 | 142 | 170 | 241 |
| mml-miR-206 | 44,390 | 778 | 1,170 |
| mml-miR-20a | 30,129 | 66,668 | 48,852 |
| mml-miR-20b | 39,640 | 91,506 | 66,043 |
| mml-miR-21 | 1,702,656 | 781,609 | 637,953 |
| mml-miR-210 | 428 | 340 | 188 |
| mml-miR-211 | 10 | 3 | 5 |
| mml-miR-212 | 25 | 38 | 54 |
| mml-miR-214 | 3,806 | 66 | 104 |
| mml-miR-215 | 605 | 665 | 56 |
| mml-miR-216a | 5 | 3 | 8 |
| mml-miR-216b | 14 | 25 | 27 |
| mml-miR-218 | 873 | 620 | 538 |
| mml-miR-219-3p | 160 | 697 | 378 |
| mml-miR-219-5p | 0 | 0 | 3 |
| mml-miR-22 | 5,264 | 687 | 573 |
| mml-miR-221 | 4,139 | 4,406 | 3,214 |
| mml-miR-222 | 4,509 | 5,133 | 3,880 |
| mml-miR-224 | 322 | 584 | 623 |
| mml-miR-23a | 1,611 | 342 | 287 |
| mml-miR-23b | 1,590 | 515 | 408 |
| mml-miR-24 | 62,264 | 28,965 | 27,577 |
| mml-miR-24* | 14 | 5 | 2 |
| mml-miR-25 | 3,372 | 6,154 | 4,694 |
| mml-miR-26a | 11,097 | 6,233 | 5,696 |
| mml-miR-26b | 2,207 | 1,132 | 860 |
| mml-miR-27a | 7,531 | 3,200 | 2,973 |
| mml-miR-27b | 35,325 | 10,535 | 11,337 |
| mml-miR-28 | 2,708 | 2,404 | 1,584 |
| mml-miR-296-3p | 3,037 | 3,478 | 1,668 |
| mml-miR-296-5p | 41 | 31 | 56 |
| mml-miR-297 | 3 | 0 | 0 |
| mml-miR-299-3p | 1,210 | 2,536 | 1,694 |
| mml-miR-299-5p | 59 | 44 | 49 |
| mml-miR-29a | 25,794 | 1,807 | 1,884 |
| mml-miR-29b | 1,292 | 120 | 130 |
| mml-miR-29c | 53 | 24 | 13 |
| mml-miR-301a | 78 | 194 | 169 |
| mml-miR-301b | 27 | 94 | 60 |
| mml-miR-302a | 6,329 | 13,051 | 11,148 |
| mml-miR-302b | 575,109 | 1,354,774 | 1,187,612 |
| mml-miR-302c | 6,608 | 18,115 | 15,677 |
| mml-miR-302d | 4,473 | 9,747 | 9,119 |
| mml-miR-30a-3p | 2,165 | 2,536 | 2,007 |
| mml-miR-30a-5p | 50,536 | 42,003 | 33,306 |
| mml-miR-30b | 220 | 306 | 252 |
| mml-miR-30c | 2,230 | 4,495 | 3,963 |
| mml-miR-30d | 81,229 | 91,133 | 72,778 |
| mml-miR-30e | 21,883 | 37,024 | 26,860 |
| mml-miR-31 | 2,968 | 403 | 324 |
| mml-miR-32 | 44 | 63 | 77 |
| mml-miR-320 | 6,355 | 10,143 | 4,996 |
| mml-miR-323-3p | 423 | 846 | 921 |
| mml-miR-323-5p | 96 | 196 | 113 |
| mml-miR-324-3p | 179 | 215 | 112 |
| mml-miR-324-5p | 92 | 85 | 98 |
| mml-miR-329 | 11 | 14 | 26 |
| mml-miR-330-3p | 256 | 764 | 540 |
| mml-miR-330-5p | 75 | 114 | 126 |
| mml-miR-331-3p | 26 | 70 | 50 |
| mml-miR-331-5p | 230 | 495 | 348 |
| mml-miR-335 | 630 | 570 | 393 |
| mml-miR-337-3p | 78 | 157 | 185 |
| mml-miR-337-5p | 3 | 10 | 4 |
| mml-miR-338-3p | 13 | 18 | 1 |
| mml-miR-338-5p | 25 | 21 | 16 |
| mml-miR-339-3p | 2,181 | 8,554 | 6,919 |
| mml-miR-339-5p | 88 | 157 | 134 |
| mml-miR-33a | 39 | 62 | 44 |
| mml-miR-33b | 3 | 0 | 1 |
| mml-miR-340 | 1,328 | 5,168 | 3,462 |
| mml-miR-342-3p | 735 | 1,612 | 1,211 |
| mml-miR-342-5p | 734 | 2,126 | 998 |
| mml-miR-346 | 0 | 2 | 0 |
| mml-miR-34a | 458 | 122 | 155 |
| mml-miR-34b | 245 | 5 | 11 |
| mml-miR-34c-3p | 7 | 12 | 8 |
| mml-miR-34c-5p | 275,328 | 19,143 | 20,965 |
| mml-miR-361-3p | 296 | 886 | 779 |
| mml-miR-361-5p | 564 | 1,820 | 1,077 |
| mml-miR-362-5p | 119 | 176 | 209 |
| mml-miR-363 | 36,515 | 72,951 | 63,735 |
| mml-miR-365 | 9 | 2 | 4 |
| mml-miR-367 | 2,627 | 2,903 | 4,066 |
| mml-miR-369-3p | 3,165 | 2,393 | 3,012 |
| mml-miR-369-5p | 348 | 142 | 146 |
| mml-miR-370 | 11,065 | 27,183 | 19,130 |
| mml-miR-371-3p | 2 | 9 | 2 |
| mml-miR-371-5p | 1,300 | 346 | 259 |
| mml-miR-372 | 2,117 | 1,220 | 1,101 |
| mml-miR-373 | 7,891 | 5,729 | 4,593 |
| mml-miR-374a | 74 | 280 | 252 |
| mml-miR-374b | 881 | 1,520 | 1,252 |
| mml-miR-375 | 311 | 310 | 304 |
| mml-miR-376a | 85 | 305 | 181 |
| mml-miR-376b | 12 | 39 | 34 |
| mml-miR-376c | 74 | 151 | 133 |
| mml-miR-377 | 227 | 27 | 29 |
| mml-miR-378 | 254,876 | 336,807 | 309,318 |
| mml-miR-379 | 250,627 | 104,692 | 86,690 |
| mml-miR-380 | 705 | 1,677 | 2,067 |
| mml-miR-381 | 7,733 | 19,167 | 12,392 |
| mml-miR-382 | 25,396 | 5,109 | 3,748 |
| mml-miR-383 | 1 | 4 | 1 |
| mml-miR-409-3p | 4,377 | 6,923 | 6,826 |
| mml-miR-409-5p | 276 | 676 | 448 |
| mml-miR-410 | 833 | 933 | 741 |
| mml-miR-411 | 9,349 | 4,645 | 3,932 |
| mml-miR-412 | 12 | 30 | 20 |
| mml-miR-421 | 321 | 733 | 484 |
| mml-miR-423-3p | 6,259 | 14,724 | 11,966 |
| mml-miR-423-5p | 8,434 | 30,295 | 18,577 |
| mml-miR-424 | 375 | 142 | 101 |
| mml-miR-425 | 2,912 | 5,620 | 4,096 |
| mml-miR-429 | 818 | 355 | 772 |
| mml-miR-431 | 142 | 100 | 110 |
| mml-miR-432 | 3,180 | 12,085 | 6,837 |
| mml-miR-433 | 785 | 126 | 120 |
| mml-miR-448 | 207 | 573 | 463 |
| mml-miR-449a | 6 | 6 | 4 |
| mml-miR-449b | 0 | 0 | 1 |
| mml-miR-450a | 112 | 15 | 20 |
| mml-miR-450b-5p | 1 | 17 | 114 |
| mml-miR-451 | 58 | 9 | 4 |
| mml-miR-452 | 1,206 | 3,159 | 2,993 |
| mml-miR-453 | 2 | 14 | 3 |
| mml-miR-454 | 124 | 329 | 266 |
| mml-miR-455-3p | 98 | 154 | 132 |
| mml-miR-455-5p | 86 | 65 | 88 |
| mml-miR-484 | 155 | 196 | 172 |
| mml-miR-485-3p | 159 | 193 | 272 |
| mml-miR-485-5p | 744 | 700 | 601 |
| mml-miR-486-3p | 17 | 21 | 11 |
| mml-miR-486-5p | 198 | 538 | 556 |
| mml-miR-487b | 107 | 197 | 138 |
| mml-miR-488 | 4 | 22 | 34 |
| mml-miR-489 | 0 | 11 | 6 |
| mml-miR-490-3p | 8 | 1 | 5 |
| mml-miR-490-5p | 2 | 9 | 0 |
| mml-miR-491-3p | 0 | 3 | 2 |
| mml-miR-491-5p | 13 | 52 | 31 |
| mml-miR-493 | 1,917 | 7,476 | 6,552 |
| mml-miR-494 | 1,180 | 1,290 | 991 |
| mml-miR-495 | 43 | 42 | 40 |
| mml-miR-496 | 24 | 15 | 8 |
| mml-miR-497 | 75 | 73 | 50 |
| mml-miR-499-3p | 1 | 0 | 2 |
| mml-miR-499-5p | 31 | 54 | 47 |
| mml-miR-500 | 6 | 10 | 13 |
| mml-miR-501 | 10 | 17 | 13 |
| mml-miR-502-3p | 44 | 89 | 83 |
| mml-miR-502-5p | 2 | 8 | 9 |
| mml-miR-503 | 79 | 214 | 138 |
| mml-miR-504 | 43 | 75 | 108 |
| mml-miR-505 | 70 | 316 | 217 |
| mml-miR-506 | 2 | 5 | 2 |
| mml-miR-508 | 0 | 0 | 2 |
| mml-miR-509 | 0 | 12 | 4 |
| mml-miR-512-3p | 12 | 19 | 19 |
| mml-miR-512-5p | 1 | 2 | 0 |
| mml-miR-514 | 1 | 0 | 2 |
| mml-miR-516a-5p | 1 | 4 | 1 |
| mml-miR-518a-3p | 2 | 2 | 0 |
| mml-miR-518a-5p | 2 | 1 | 0 |
| mml-miR-518b | 0 | 1 | 1 |
| mml-miR-518f | 3 | 1 | 0 |
| mml-miR-519c | 0 | 0 | 2 |
| mml-miR-520d-3p | 2 | 2 | 0 |
| mml-miR-520d-5p | 2 | 1 | 0 |
| mml-miR-523a | 0 | 0 | 1 |
| mml-miR-523b | 0 | 0 | 2 |
| mml-miR-532-3p | 27 | 9 | 17 |
| mml-miR-532-5p | 1,468 | 1,032 | 1,145 |
| mml-miR-539 | 131 | 98 | 114 |
| mml-miR-542-3p | 1,848 | 662 | 698 |
| mml-miR-542-5p | 96 | 53 | 41 |
| mml-miR-544 | 3 | 0 | 0 |
| mml-miR-548b | 1 | 0 | 1 |
| mml-miR-550 | 18 | 15 | 18 |
| mml-miR-551a | 12 | 50 | 17 |
| mml-miR-552 | 0 | 0 | 3 |
| mml-miR-572 | 0 | 1 | 0 |
| mml-miR-576-3p | 42 | 113 | 49 |
| mml-miR-576-5p | 6 | 8 | 14 |
| mml-miR-577 | 1,078 | 2,037 | 1,902 |
| mml-miR-578 | 2 | 0 | 0 |
| mml-miR-580 | 3 | 8 | 1 |
| mml-miR-581 | 1 | 4 | 4 |
| mml-miR-582-3p | 21 | 68 | 65 |
| mml-miR-582-5p | 7 | 4 | 3 |
| mml-miR-584 | 42 | 89 | 45 |
| mml-miR-589 | 0 | 11 | 2 |
| mml-miR-590-3p | 101 | 136 | 109 |
| mml-miR-590-5p | 2 | 4 | 4 |
| mml-miR-592 | 5 | 3 | 3 |
| mml-miR-598 | 1,820 | 3,455 | 3,358 |
| mml-miR-605 | 0 | 0 | 2 |
| mml-miR-607 | 0 | 2 | 0 |
| mml-miR-615-3p | 54 | 1 | 1 |
| mml-miR-615-5p | 35 | 2 | 0 |
| mml-miR-616 | 0 | 2 | 3 |
| mml-miR-624 | 6 | 18 | 2 |
| mml-miR-628-3p | 3 | 5 | 3 |
| mml-miR-628-5p | 241 | 487 | 364 |
| mml-miR-632 | 1 | 0 | 0 |
| mml-miR-636 | 2 | 9 | 7 |
| mml-miR-639 | 0 | 1 | 0 |
| mml-miR-642 | 0 | 1 | 0 |
| mml-miR-652 | 1,878 | 1,338 | 739 |
| mml-miR-653 | 9 | 16 | 7 |
| mml-miR-654-3p | 775 | 2,060 | 1,816 |
| mml-miR-654-5p | 494 | 1,830 | 900 |
| mml-miR-656 | 6 | 30 | 25 |
| mml-miR-660 | 35 | 52 | 64 |
| mml-miR-663 | 0 | 0 | 1 |
| mml-miR-668 | 71 | 73 | 30 |
| mml-miR-671-3p | 46 | 36 | 20 |
| mml-miR-671-5p | 2,561 | 4,831 | 2,490 |
| mml-miR-675 | 0 | 1 | 0 |
| mml-miR-7 | 4,298 | 13,811 | 9,921 |
| mml-miR-758 | 73 | 273 | 172 |
| mml-miR-767-3p | 5 | 17 | 7 |
| mml-miR-767-5p | 945 | 5,125 | 3,895 |
| mml-miR-770-5p | 1 | 3 | 2 |
| mml-miR-874 | 108 | 323 | 277 |
| mml-miR-876-5p | 0 | 1 | 0 |
| mml-miR-877 | 3,666 | 6,424 | 4,897 |
| mml-miR-885-3p | 1 | 12 | 4 |
| mml-miR-885-5p | 0 | 0 | 3 |
| mml-miR-886-5p | 1,522 | 557 | 497 |
| mml-miR-887 | 202 | 660 | 402 |
| mml-miR-889 | 1,349 | 3,709 | 3,708 |
| mml-miR-9 | 14,873 | 48,766 | 36,191 |
| mml-miR-92a | 24,114 | 43,627 | 38,134 |
| mml-miR-92b | 3,961 | 9,143 | 8,887 |
| mml-miR-93 | 93,982 | 183,499 | 137,587 |
| mml-miR-933 | 1 | 0 | 0 |
| mml-miR-937 | 5 | 5 | 1 |
| mml-miR-939 | 3 | 27 | 7 |
| mml-miR-940 | 0 | 8 | 1 |
| mml-miR-942 | 382 | 1,167 | 967 |
| mml-miR-95 | 71 | 143 | 169 |
| mml-miR-96 | 415 | 718 | 744 |
| mml-miR-98 | 968 | 106 | 89 |
| mml-miR-99a | 1,927 | 130 | 108 |
| mml-miR-99b | 343,414 | 154,591 | 142,761 |

Table S2. The novel miRNAs shared by the three rESC lines

|  |  |  |  |  | IVF1.2 | | | IVF3.2 | | | IVF3.3 | | |
| --- | --- | --- | --- | --- | --- | --- | --- | --- | --- | --- | --- | --- | --- |
| location | mfe | sequence(5p) | sequence(3p) | Annotation | counts | counts(5p) | counts(3p) | counts | counts(5p) | counts(3p) | counts | counts(5p) | counts(3p) |
| chr1:158332926:158333000:- | -49.4 | GTTGGGACAAGAGAACGGTCTT | - | mir-3122 | 252 | 252 | - | 371 | 371 | - | 214 | 214 | - |
| chr1:17505345:17505431:+ | -28.9 | - | AATGGACTTGGAGTCAGAAGGC |  | 50 | - | 50 | 52 | - | 52 | 61 | - | 61 |
| chr1:177443834:177443915:- | -45.9 | - | ATTTATGAACAGGCAGGAAGAA |  | 105 | - | 105 | 88 | - | 88 | 54 | - | 54 |
| chr1:177443834:177443915:+ | -42.3 | - | ATTTATGAACAGGCAGGAAGAA |  | 105 | - | 105 | 88 | - | 88 | 54 | - | 54 |
| chr1:205580537:205580616:- | -22 | TATGGAGGTCTCTGTCTGGCT | TCTGATCGTTCCCCTCCATACA | mir-1843 | 251 | 124 | 127 | 333 | 159 | 174 | 345 | 152 | 193 |
| chr1:212022320:212022395:- | -34.6 | AGTGTACTTCCTGAGGCCTCTGG | - |  | 75 | 75 | - | 291 | 291 | - | 231 | 231 | - |
| chr1:5313874:5313948:+ | -38.5 | ACTGCTGCCCTGATAGTCGG | - |  | 40 | 40 | - | 129 | 129 | - | 84 | 84 | - |
| chr1:6543837:6543915:- | -45.3 | GTTAATCATTGGTCTCTGTGTCC | - |  | 89 | 89 | - | 456 | 456 | - | 382 | 382 | - |
| chr1:70976129:70976208:- | -46.8 | - | TGATGGGTGAATTTGTAGAAGG | mir-1262 | 2557 | - | 2557 | 2267 | - | 2267 | 2150 | - | 2150 |
| chr10:32419797:32419874:- | -33.6 | TGCAGCCAGCAGTGGGACCTAAG | - |  | 48 | 48 | - | 143 | 143 | - | 75 | 75 | - |
| chr10:84714582:84714655:- | -35.3 | TCTGAAAGAGCAGTTGGTGTTT | - |  | 127 | 127 | - | 223 | 223 | - | 138 | 138 | - |
| chr10:87993356:87993435:+ | -39.8 | TCGAGGGGCGTCGGGGCCAGGGAAGC | TCTCTGACACACGCCCTCCTGC |  | 220 | 146 | 74 | 392 | 296 | 96 | 262 | 128 | 134 |
| chr10:90328879:90328966:+ | -21.4 | - | GTTTGATGATGTTGCCTGATG |  | 146 | - | 146 | 111 | - | 111 | 117 | - | 117 |
| chr11:105706113:105706195:+ | -57.8 | - | TGTGGGACCTCTGGCCTTGGC | mir-3922 | 192 | - | 192 | 250 | - | 250 | 248 | - | 248 |
| chr11:98713141:98713223:+ | -32.5 | GACTCTAGCTGCCAAAGGCGCT | - | mir-1251 | 40 | 40 | - | 97 | 97 | - | 59 | 59 | - |
| chr12:4550806:4550896:- | -27 | AAGAACCAAGAATGGGCTGC | - |  | 41 | 41 | - | 98 | 98 | - | 85 | 85 | - |
| chr13:111712248:111712342:- | -24.2 | AAGAACCAAGAATGGGCTGC | - |  | 41 | 41 | - | 98 | 98 | - | 85 | 85 | - |
| chr13:118015549:118015620:- | -45.9 | - | TTGGGACCTCACTCACTCACGC |  | 84 | - | 84 | 195 | - | 195 | 146 | - | 146 |
| chr13:75089538:75089619:+ | -50.3 | TCCTGGGCTTTGGCAGACAGCT | - |  | 95 | 95 | - | 179 | 179 | - | 109 | 109 | - |
| chr15:1930020:1930096:+ | -48.7 | TGAGGGCCGGGGGCTGGGAACGG | - |  | 123 | 123 | - | 124 | 124 | - | 67 | 67 | - |
| chr16:11829804:11829901:+ | -38.9 | TGCGGGGCTAGGGCTAACAGCA | CTGTTGCCACTAACCTCAACC | mir-744 | 11522 | 11447 | 75 | 24878 | 24797 | 81 | 14275 | 14171 | 104 |
| chr16:18891345:18891423:- | -50.2 | - | TTTCCGGCTCGCGTGGGTGTGT | mir-1180 | 30 | - | 30 | 133 | - | 133 | 77 | - | 77 |
| chr16:70301595:70301674:- | -34.2 | - | GACTGTGCTCCCAAGATAACTTTT |  | 121 | - | 121 | 120 | - | 120 | 133 | - | 133 |
| chr17:90323129:90323209:- | -45 | AGGACTGCTGGAGGACCGCAGAG | - |  | 94 | 94 | - | 192 | 192 | - | 187 | 187 | - |
| chr18:15935565:15935655:+ | -55.6 | - | CGGGCTGTCCGGAGGGGTCGGC |  | 35 | - | 35 | 115 | - | 115 | 67 | - | 67 |
| chr19:61698713:61698794:+ | -34.9 | AGGGGCATAGGCTTGAGCAGAGG | - |  | 30 | 30 | - | 101 | 101 | - | 52 | 52 | - |
| chr2:188929960:188930030:- | -39.4 | - | TGCAGCCTGGGTGGAGCCTGAC |  | 46 | - | 46 | 176 | - | 176 | 89 | - | 89 |
| chr2:40118453:40118533:- | -29.3 | GAGAGATCAGAGGCGCAGAGT | - |  | 2385 | 2385 | - | 5990 | 5990 | - | 4208 | 4208 | - |
| chr2:80519804:80519882:+ | -49.6 | - | AATTCCCTTATGGATAATCTGG | mir-3938 | 138 | - | 138 | 227 | - | 227 | 104 | - | 104 |
| chr20:87818403:87818477:+ | -45.3 | TGTGGCTGTGTGCTGAGGGTC | - |  | 2817 | 2817 | - | 4722 | 4722 | - | 2633 | 2633 | - |
| chr3:106443245:106443327:+ | -31.5 | - | CATGCTAGAACAGAAAGAATGGG | mir-3146 | 37 | - | 37 | 120 | - | 120 | 61 | - | 61 |
| chr3:176724091:176724162:- | -42.1 | CTGTGGTTCCTGTATGAAGACA | - |  | 1319 | 1319 | - | 2140 | 2140 | - | 2251 | 2251 | - |
| chr3:192875970:192876064:+ | -18.3 | TGGCTGTGATGTTTGACTGAGT | - |  | 73 | 73 | - | 97 | 97 | - | 85 | 85 | - |
| chr3:2598731:2598807:- | -45.9 | TGCGGGCTGTCAGCGCAAAGGATG | - |  | 42 | 42 | - | 374 | 374 | - | 207 | 207 | - |
| chr3:38674232:38674306:+ | -38.3 | CCGGCCATGCACCTCTGCCTTG | - |  | 102 | 102 | - | 226 | 226 | - | 256 | 256 | - |
| chr3:80708283:80708358:- | -25.7 | GTTGAATGATGTGAGCTGACC | - |  | 61 | 61 | - | 135 | 135 | - | 94 | 94 | - |
| chr3:82226017:82226111:- | -40.9 | - | TGGACTCCTCTGCCGATGCTCAGC |  | 180 | - | 180 | 148 | - | 148 | 120 | - | 120 |
| chr4:125407861:125407939:+ | -27.8 | - | TATTTTGAGTGTTTGGAATTGA | mir-3145 | 35 | - | 35 | 36 | - | 36 | 31 | - | 31 |
| chr4:128869861:128869951:+ | -19.9 | - | GAAAATGATGAGTAGTGACTGATG | mir-3622 | 72 | - | 72 | 243 | - | 243 | 73 | - | 73 |
| chr4:26037916:26037996:- | -20.2 | GTCGCAAAGAGAGCTATAACCAC | - |  | 36 | 36 | - | 76 | 76 | - | 52 | 52 | - |
| chr5:102714456:102714543:+ | -29.2 | - | GGAGAATGATGTAAACTGACG |  | 476 | - | 476 | 607 | - | 607 | 490 | - | 490 |
| chr5:144708317:144708405:- | -41.4 | - | AAGAGCTTTTGGGAATTCAGGTAG | mir-3140 | 151 | - | 151 | 358 | - | 358 | 200 | - | 200 |
| chr6:115873207:115873278:+ | -29.3 | TGTGATGATGACAGAACTGAGC | - |  | 306 | 306 | - | 826 | 826 | - | 513 | 513 | - |
| chr6:167320742:167320828:+ | -36.1 | TAAGAAATAGGTCATTAACAGTAG | - |  | 33 | 33 | - | 103 | 103 | - | 57 | 57 | - |
| chr6:68319118:68319194:+ | -44.9 | TAACATAATAGTGTGGACTGA | - |  | 31 | 31 | - | 47 | 47 | - | 40 | 40 | - |
| chr6:72885052:72885132:+ | -40.3 | ATGGGGACAGTTTTTGTAGTACA | - |  | 35 | 35 | - | 129 | 129 | - | 81 | 81 | - |
| chr7:164327476:164327555:+ | -33.5 | - | ATATACAGGGGGAGACTCTTAT | mir-1185-1 | 52 | - | 52 | 176 | - | 176 | 105 | - | 105 |
| chr7:164328696:164328775:+ | -33.5 | - | ATATACAGGGGGAGACTCTTAT | mir-1185-2 | 52 | - | 52 | 176 | - | 176 | 105 | - | 105 |
| chr7:164844819:164844896:- | -36.6 | - | CGGGAACGTCGAGACTGGAGC | mir-1247 | 127 | - | 127 | 131 | - | 131 | 32 | - | 32 |
| chr7:53825925:53826013:+ | -47.7 | - | TTGTTTTTTATTCTGAGTGACA |  | 75 | - | 75 | 234 | - | 234 | 245 | - | 245 |
| chr7:86188751:86188827:- | -45.8 | TGGACTTACTGCTGCCAGGGGA | - |  | 111 | 111 | - | 252 | 252 | - | 148 | 148 | - |
| chr8:12369632:12369713:+ | -53.4 | TGCCTGCTGTGGGGGCCCCGGC | - |  | 35 | 35 | - | 79 | 79 | - | 72 | 72 | - |
| chr8:139852435:139852527:+ | -28.4 | AAGAACCAAGAATGGGCTGC | - |  | 41 | 41 | - | 98 | 98 | - | 85 | 85 | - |
| chr8:96489944:96490019:- | -26.2 | ACTGGACTTGGAGTCAGAAGAC | - |  | 197 | 197 | - | 96 | 96 | - | 103 | 103 | - |
| chr9:103084702:103084783:- | -32.8 | TCGACCGGACCTCGACCGGCTCG | ACTCGGCGTGGCGTCGGTCGTGG | mir-1307 | 2227 | 291 | 1936 | 4847 | 872 | 3975 | 3974 | 944 | 3030 |
| chr9:46683312:46683388:+ | -27.24 | - | CTCAATGTGGGATCCGGCTGC |  | 42 | - | 42 | 59 | - | 59 | 38 | - | 38 |
| chr9:73887081:73887164:+ | -49.3 | TTAGGGCCCTGGCTCCATCTCC | - | mir-1296 | 33 | 33 | - | 123 | 123 | - | 53 | 53 | - |
| chrX:113236410:113236505:+ | -43.4 | TTCATTCGGCTGTCCAGATGTA | CATCTGGGCAACTGACTGAACT | mir-1298 | 276 | 246 | 30 | 991 | 893 | 98 | 910 | 852 | 58 |
| chrX:113271154:113271232:+ | -33.8 | TGAGTACCGCCATGTCTGTTGGG | - | mir-1911 | 88 | 88 | - | 843 | 843 | - | 473 | 473 | - |
| chrX:131717087:131717164:+ | -22.4 | - | TGGGCCTTACCCTGAGTAGAGC |  | 92 | - | 92 | 386 | - | 386 | 294 | - | 294 |
| chrX:20545557:20545634:- | -42 | TGGTGAAAGCTGGGAATGCAGA | - |  | 315 | 315 | - | 253 | 253 | - | 181 | 181 | - |
| chrX:68990098:68990174:+ | -35.3 | - | CCGTCCTAAGGTTGTTGAGTT | mir-676 | 46 | - | 46 | 206 | - | 206 | 206 | - | 206 |

Table S3. Over-representation of the predicted miRNA targets for both conserved and non-conserved patterns based on the TargetScan program in the KEGG pathway

| **Conserved pattern** | | | | | |
| --- | --- | --- | --- | --- | --- |
| **Category** | **Term** | **Count** | **%** | **PValue** | **Benjamini** |
| KEGG_PATHWAY | hsa05200:Pathways in cancer | 245 | 2.669427 | 4.80E-22 | 9.47E-20 |
| KEGG_PATHWAY | hsa04360:Axon guidance | 106 | 1.154936 | 1.95E-14 | 1.92E-12 |
| KEGG_PATHWAY | hsa04010:MAPK signaling pathway | 190 | 2.070168 | 1.79E-13 | 1.18E-11 |
| KEGG_PATHWAY | hsa04310:Wnt signaling pathway | 118 | 1.285683 | 4.61E-13 | 2.27E-11 |
| KEGG_PATHWAY | hsa04510:Focal adhesion | 147 | 1.601656 | 4.93E-12 | 1.94E-10 |
| KEGG_PATHWAY | hsa04810:Regulation of actin cytoskeleton | 155 | 1.688821 | 7.97E-12 | 2.62E-10 |
| KEGG_PATHWAY | hsa04144:Endocytosis | 135 | 1.470909 | 2.85E-11 | 8.02E-10 |
| KEGG_PATHWAY | hsa04350:TGF-beta signaling pathway | 72 | 0.784485 | 2.98E-10 | 7.34E-09 |
| KEGG_PATHWAY | hsa05210:Colorectal cancer | 68 | 0.740902 | 6.07E-09 | 1.33E-07 |
| KEGG_PATHWAY | hsa04520:Adherens junction | 63 | 0.686424 | 1.14E-08 | 2.25E-07 |
| KEGG_PATHWAY | hsa04722:Neurotrophin signaling pathway | 92 | 1.002397 | 2.55E-08 | 4.57E-07 |
| KEGG_PATHWAY | hsa04910:Insulin signaling pathway | 97 | 1.056875 | 1.41E-07 | 2.31E-06 |
| KEGG_PATHWAY | hsa05211:Renal cell carcinoma | 56 | 0.610155 | 3.69E-07 | 5.59E-06 |
| KEGG_PATHWAY | hsa05220:Chronic myeloid leukemia | 59 | 0.642842 | 4.74E-07 | 6.67E-06 |
| KEGG_PATHWAY | hsa05215:Prostate cancer | 67 | 0.730007 | 1.17E-06 | 1.53E-05 |
| KEGG_PATHWAY | hsa05222:Small cell lung cancer | 63 | 0.686424 | 3.24E-06 | 3.75E-05 |
| KEGG_PATHWAY | hsa04530:Tight junction | 93 | 1.013293 | 3.14E-06 | 3.86E-05 |
| KEGG_PATHWAY | hsa04916:Melanogenesis | 71 | 0.773589 | 9.84E-06 | 1.08E-04 |
| KEGG_PATHWAY | hsa04666:Fc gamma R-mediated phagocytosis | 68 | 0.740902 | 1.75E-05 | 1.82E-04 |
| KEGG_PATHWAY | hsa05212:Pancreatic cancer | 54 | 0.588363 | 1.92E-05 | 1.90E-04 |
| KEGG_PATHWAY | hsa05218:Melanoma | 53 | 0.577468 | 2.92E-05 | 2.62E-04 |
| KEGG_PATHWAY | hsa04120:Ubiquitin mediated proteolysis | 92 | 1.002397 | 2.85E-05 | 2.67E-04 |
| KEGG_PATHWAY | hsa04660:T cell receptor signaling pathway | 75 | 0.817171 | 3.24E-05 | 2.78E-04 |
| KEGG_PATHWAY | hsa05412:Arrhythmogenic right ventricular cardiomyopathy (ARVC) | 55 | 0.599259 | 8.30E-05 | 6.81E-04 |
| KEGG_PATHWAY | hsa04720:Long-term potentiation | 50 | 0.544781 | 9.95E-05 | 7.54E-04 |
| KEGG_PATHWAY | hsa05214:Glioma | 47 | 0.512094 | 9.59E-05 | 7.55E-04 |
| KEGG_PATHWAY | hsa05217:Basal cell carcinoma | 42 | 0.457616 | 1.06E-04 | 7.73E-04 |
| KEGG_PATHWAY | hsa04150:mTOR signaling pathway | 40 | 0.435825 | 1.25E-04 | 8.79E-04 |
| KEGG_PATHWAY | hsa04020:Calcium signaling pathway | 111 | 1.209414 | 2.14E-04 | 0.00145 |
| KEGG_PATHWAY | hsa05014:Amyotrophic lateral sclerosis (ALS) | 40 | 0.435825 | 2.49E-04 | 0.001637 |
| KEGG_PATHWAY | hsa04012:ErbB signaling pathway | 60 | 0.653737 | 3.14E-04 | 0.001993 |
| KEGG_PATHWAY | hsa04330:Notch signaling pathway | 36 | 0.392242 | 3.53E-04 | 0.002174 |
| KEGG_PATHWAY | hsa04070:Phosphatidylinositol signaling system | 52 | 0.566572 | 4.37E-04 | 0.002607 |
| KEGG_PATHWAY | hsa05221:Acute myeloid leukemia | 42 | 0.457616 | 7.05E-04 | 0.004078 |
| KEGG_PATHWAY | hsa04540:Gap junction | 60 | 0.653737 | 7.79E-04 | 0.004375 |
| KEGG_PATHWAY | hsa04930:Type II diabetes mellitus | 35 | 0.381347 | 0.001041 | 0.005684 |
| KEGG_PATHWAY | hsa04664:Fc epsilon RI signaling pathway | 53 | 0.577468 | 0.001297 | 0.006706 |
| KEGG_PATHWAY | hsa05223:Non-small cell lung cancer | 39 | 0.424929 | 0.001263 | 0.006706 |
| KEGG_PATHWAY | hsa05120:Epithelial cell signaling in Helicobacter pylori infection | 47 | 0.512094 | 0.001537 | 0.007738 |
| KEGG_PATHWAY | hsa04512:ECM-receptor interaction | 56 | 0.610155 | 0.001799 | 0.008829 |
| KEGG_PATHWAY | hsa04920:Adipocytokine signaling pathway | 46 | 0.501199 | 0.002155 | 0.010313 |
| KEGG_PATHWAY | hsa05414:Dilated cardiomyopathy | 60 | 0.653737 | 0.002601 | 0.012143 |
| KEGG_PATHWAY | hsa05213:Endometrial cancer | 37 | 0.403138 | 0.002664 | 0.012145 |
| KEGG_PATHWAY | hsa04115:p53 signaling pathway | 46 | 0.501199 | 0.003365 | 0.014648 |
| KEGG_PATHWAY | hsa04912:GnRH signaling pathway | 63 | 0.686424 | 0.003297 | 0.014675 |
| KEGG_PATHWAY | hsa05110:Vibrio cholerae infection | 39 | 0.424929 | 0.003564 | 0.014855 |
| KEGG_PATHWAY | hsa04340:Hedgehog signaling pathway | 39 | 0.424929 | 0.003564 | 0.014855 |
| KEGG_PATHWAY | hsa04670:Leukocyte transendothelial migration | 74 | 0.806276 | 0.003524 | 0.015007 |
| KEGG_PATHWAY | hsa04370:VEGF signaling pathway | 49 | 0.533885 | 0.006695 | 0.027195 |
| KEGG_PATHWAY | hsa05410:Hypertrophic cardiomyopathy (HCM) | 54 | 0.588363 | 0.009644 | 0.037463 |
| KEGG_PATHWAY | hsa05216:Thyroid cancer | 22 | 0.239704 | 0.009514 | 0.037702 |
| KEGG_PATHWAY | hsa04730:Long-term depression | 45 | 0.490303 | 0.010134 | 0.03858 |
| KEGG_PATHWAY | hsa00534:Heparan sulfate biosynthesis | 20 | 0.217912 | 0.011648 | 0.043415 |

| **Non-conserved pattern** | | | | | |
| --- | --- | --- | --- | --- | --- |
| **Category** | **Term** | **Count** | **%** | **PValue** | **Benjamini** |
| KEGG_PATHWAY | hsa05200:Pathways in cancer | 312 | 1.907557 | 6.85E-09 | 1.36E-06 |
| KEGG_PATHWAY | hsa04510:Focal adhesion | 193 | 1.179995 | 1.19E-06 | 1.19E-04 |
| KEGG_PATHWAY | hsa04310:Wnt signaling pathway | 146 | 0.892639 | 9.86E-06 | 6.54E-04 |
| KEGG_PATHWAY | hsa04910:Insulin signaling pathway | 130 | 0.794815 | 7.38E-05 | 0.002934468 |
| KEGG_PATHWAY | hsa04722:Neurotrophin signaling pathway | 120 | 0.733676 | 7.38E-05 | 0.003663526 |
| KEGG_PATHWAY | hsa04115:p53 signaling pathway | 68 | 0.41575 | 1.90E-04 | 0.006274403 |
| KEGG_PATHWAY | hsa05120:Epithelial cell signaling in Helicobacter pylori infection | 68 | 0.41575 | 1.90E-04 | 0.006274403 |
| KEGG_PATHWAY | hsa04020:Calcium signaling pathway | 166 | 1.014918 | 2.22E-04 | 0.006293825 |
| KEGG_PATHWAY | hsa04144:Endocytosis | 173 | 1.057716 | 2.60E-04 | 0.006443833 |
| KEGG_PATHWAY | hsa04514:Cell adhesion molecules (CAMs) | 126 | 0.77036 | 3.68E-04 | 0.008103596 |
| KEGG_PATHWAY | hsa05220:Chronic myeloid leukemia | 74 | 0.452433 | 4.70E-04 | 0.00847303 |
| KEGG_PATHWAY | hsa04360:Axon guidance | 123 | 0.752018 | 5.18E-04 | 0.008555821 |
| KEGG_PATHWAY | hsa04350:TGF-beta signaling pathway | 85 | 0.519687 | 4.69E-04 | 0.009284048 |
| KEGG_PATHWAY | hsa04060:Cytokine-cytokine receptor interaction | 241 | 1.473465 | 7.30E-04 | 0.010330271 |
| KEGG_PATHWAY | hsa05212:Pancreatic cancer | 71 | 0.434091 | 7.15E-04 | 0.01088805 |
| KEGG_PATHWAY | hsa04210:Apoptosis | 84 | 0.513573 | 0.001913 | 0.01985782 |
| KEGG_PATHWAY | hsa04010:MAPK signaling pathway | 244 | 1.491807 | 0.001882 | 0.020612363 |
| KEGG_PATHWAY | hsa04660:T cell receptor signaling pathway | 103 | 0.629738 | 0.001791 | 0.020762192 |
| KEGG_PATHWAY | hsa04666:Fc gamma R-mediated phagocytosis | 91 | 0.556371 | 0.002515 | 0.021553072 |
| KEGG_PATHWAY | hsa04110:Cell cycle | 118 | 0.721448 | 0.00231 | 0.021677776 |
| KEGG_PATHWAY | hsa05014:Amyotrophic lateral sclerosis (ALS) | 53 | 0.32404 | 0.001787 | 0.022001499 |
| KEGG_PATHWAY | hsa05214:Glioma | 62 | 0.379066 | 0.002461 | 0.02203964 |
| KEGG_PATHWAY | hsa00230:Purine metabolism | 143 | 0.874297 | 0.002249 | 0.0221542 |
| KEGG_PATHWAY | hsa04621:NOD-like receptor signaling pathway | 61 | 0.372952 | 0.002817 | 0.022204829 |
| KEGG_PATHWAY | hsa04512:ECM-receptor interaction | 81 | 0.495231 | 0.00275 | 0.022576109 |
| KEGG_PATHWAY | hsa05210:Colorectal cancer | 81 | 0.495231 | 0.00275 | 0.022576109 |
| KEGG_PATHWAY | hsa05222:Small cell lung cancer | 81 | 0.495231 | 0.00275 | 0.022576109 |
| KEGG_PATHWAY | hsa04670:Leukocyte transendothelial migration | 112 | 0.684764 | 0.001756 | 0.02305236 |
| KEGG_PATHWAY | hsa05211:Renal cell carcinoma | 68 | 0.41575 | 0.004206 | 0.031742073 |
| KEGG_PATHWAY | hsa04062:Chemokine signaling pathway | 172 | 1.051602 | 0.005286 | 0.034547265 |
| KEGG_PATHWAY | hsa05215:Prostate cancer | 85 | 0.519687 | 0.004972 | 0.034808103 |
| KEGG_PATHWAY | hsa04916:Melanogenesis | 94 | 0.574713 | 0.004815 | 0.034949843 |
| KEGG_PATHWAY | hsa03040:Spliceosome | 118 | 0.721448 | 0.005277 | 0.035657263 |
| KEGG_PATHWAY | hsa04012:ErbB signaling pathway | 83 | 0.507459 | 0.006213 | 0.038013983 |
| KEGG_PATHWAY | hsa04920:Adipocytokine signaling pathway | 65 | 0.397408 | 0.006103 | 0.038533141 |
| KEGG_PATHWAY | hsa04662:B cell receptor signaling pathway | 72 | 0.440205 | 0.00793 | 0.045532559 |
| KEGG_PATHWAY | hsa04810:Regulation of actin cytoskeleton | 196 | 1.198337 | 0.00793 | 0.046874319 |
| KEGG_PATHWAY | hsa04070:Phosphatidylinositol signaling system | 71 | 0.434091 | 0.008894 | 0.04952719 |

Table S4. PCR primers and condition for RT-PCR

| Genes | Primer sequences | Annealing temp | Productes | cycles |
| --- | --- | --- | --- | --- |
| Sox2 | 5′-CCCCCGGCGGCAATAGCA-3′ | 58℃ | 448bp | 30 |
|  | 5′-TCGGCGCCGGGGAGATACAT-3′ |  |  |  |
| Rex-1 | 5′-GCGTACGCAAATTAAAGTCCAGA-3′ | 56℃ | 350bp | 30 |
|  | 5′-CAGCATCCTAAACAGCTCGCAGAAT-3′ |  |  |  |
